# Probiotic attributes and safety profile of AKM Lab-01: a novel *Akkermansia muciniphila* strain combating obesity and metabolic disorders in diet-induced obese mice

**DOI:** 10.3389/fmicb.2025.1627870

**Published:** 2026-01-06

**Authors:** Baojia Huang, Yibo Xian, Wenbin Xue, Zilun Pu, Ping Kong, Peifen Li, Yingying Zhao, Lihong Tai, Zhipeng Chen, Zhou Lan, Hong-Wei Liu, Xianzhi Jiang, Amanda Juan Chen

**Affiliations:** 1 Moon (Guangzhou) Biotech Co. Ltd., Guangzhou, Guangdong, China; 2 State Key Laboratory of Microbial Diversity and Innovative Utilization, Institute of Microbiology, Chinese Academy of Sciences, Beijing, China

**Keywords:** *Akkermansia muciniphila* AKM Lab-01, comparative genome, anti-obesity, metabolic disorders, probiotic, safety

## Abstract

Obesity is a global health crisis associated with numerous chronic diseases. Recent studies highlight the role of gut microbiota, particularly *Akkermansia muciniphila* (*A. muciniphila*), in regulating metabolism and improving obesity-related disorders. In this study, we isolated 222 strains of *Akkermansia* from healthy human feces. Using a genomic dataset of 26 selected strains (from our isolation) plus 97 from NCBI, phylogenetic analysis revealed that there are eight *Akkermansia* species including two major clades: *A. muciniphila* and *Akkermansia massiliensis*, along with two potential novel candidate species. One strain, designated AKM Lab-01, demonstrated significant efficacy in reducing weight gain in a high-fat diet (HFD)-induced obese mouse model, with a 10% weight reduction observed after 4 weeks. Metabolic parameters (e.g., blood glucose, lipids, and insulin resistance) and liver/kidney function were also improved. AKM Lab-01 exhibited strong probiotic properties, including pH and bile salt tolerance, high auto aggregation capacity, and antibiotic sensitivity. Further investigations revealed that both live and processed forms (pasteurized and powdered) of AKM Lab-01 effectively mitigated obesity and associated metabolic disorders in diet-induce obese mice. A 90-day dietary exposure study in mice demonstrated no adverse effects at doses up to 8 × 10^10^ total florescent unit (TFU) a day. In accordance with EFSA guidelines and applying an uncertainty factor of 200, a daily intake of 1 × 10^12^ TFU is deemed safe for human consumption. Collectively, these findings highlight the potential of AKM Lab-01 as a promising probiotic for the prevention and treatment of obesity and related metabolic diseases.

## 1 Introduction

Obesity has become a global health issue, severely affecting human quality of life and lifespan. According to a study published in The Lancet, the global prevalence of adult obesity increased by 3.5 times from 1990 to 2022, while the obesity rate among children and adolescents grew by more than fourfold (Collaboration, 2024). In 2022, the number of obese individuals worldwide exceeded 1 billion, including approximately 160 million children and adolescents and 880 million adults (Collaboration, 2024). Obesity not only increases the risk of chronic diseases such as cardiovascular diseases, diabetes, and cancer (Wilcox et al., 2024), but also negatively impacts mental health by increasing the risk of depression and anxiety (Xia et al., 2021; Wang et al., 2024). Moreover, the economic impact of the obesity epidemic is significant. The World Health Organization predicts that if no measures are taken, the annual cost of overweight and obesity will reach US$3 trillion by 2030 and exceed US$18 trillion by 2060 (World Health Organization, 2024). Therefore, obesity has become a major challenge in the field of global public health that urgently needs to be addressed. In recent years, the interaction between the gut microbiota and host metabolism has gradually become a research hotspot. With the development of multi-omics technologies, especially the widespread application of metagenomics, metabolomics, and transcriptomics, scientists have achieved a more comprehensive understanding of the composition, function, and complex relationship between the gut microbiota and host metabolism (Fan and Pedersen, 2021). Studies have found that the gut microbiota regulates host energy metabolism, immune response, and neurosignal transmission through the production of bioactive substances such as short-chain fatty acids (SCFAs), secondary bile acids, and tryptophan metabolites (Koh et al., 2016). For example, SCFAs can promote the secretion of incretins by activating G protein-coupled receptors, thereby improving insulin sensitivity (Canfora et al., 2019). Additionally, gut microbiota also affects host lipid metabolism and cholesterol levels by regulating bile acid metabolism (Collins et al., 2023). These findings not only reveal the key role of the gut microbiota in maintaining host metabolic homeostasis but also provide a theoretical basis for the development of microbiota-based disease intervention strategies (Van Hul and Cani, 2023).

*Akkermansia muciniphila*, a mucin-degrading bacterium, was first isolated from human feces in 2004 (Derrien et al., 2004). It is the only known gut microbe that can utilize mucin as its sole carbon and nitrogen source (Ioannou et al., 2025). To date, scientific literature has documented over 1,500 peer-reviewed publications concerning the investigation of *A. muciniphila*. *A. muciniphila* has attracted attention for its potential role in regulating host energy metabolism and improving obesity (Fan and Pedersen, 2021; Cani et al., 2022; Jian et al., 2023). In clinical cohort studies, *A. muciniphila* abundance has been found to be negatively correlated with the degree of obesity, indicating that its levels are relatively reduced in the gut microbiota of obese individuals (Dao et al., 2016). In multiple animal experiments, researchers have found that supplementation with in HFD-induced obese mice significantly reduces body weight, decreases fat accumulation, and improve glucose tolerance (Schneeberger et al., 2015; Cani et al., 2022). In a randomized, double-blind, placebo-controlled human intervention study, overweight and obese individuals with insulin resistance were given a diet supplemented with live or pasteurized *A. muciniphila* for 3 months. The results showed that pasteurized *A. muciniphila* intervention effectively improved insulin sensitivity, reduced insulin levels, plasma cholesterol, and body fat distribution (Depommier et al., 2019). With the rapid development of multi-omics technologies, scientists have gained a deeper understanding of the physiological functions of *A. muciniphila* and its interactions with host metabolism (Fan and Pedersen, 2021; Cani et al., 2022; Jian et al., 2023). Studies have shown that *A. muciniphila* can regulate host energy metabolism through multiple mechanisms, contributing to beneficial effects on obesity (Plovier et al., 2017). Specifically, *A. muciniphila* can regulate the thickness of the intestinal mucus layer, improve gut barrier function, reduce the entry of endotoxins into the bloodstream, and thereby alleviate systemic inflammatory responses (Everard et al., 2013; Plovier et al., 2017; Wang et al., 2020). This inflammatory response is one of the important factors in the development of obesity and related metabolic diseases (Cani et al., 2008). Very recently, Sun et al. demonstrated that the hypo-acylated rough-type lipopolysaccharide (ALPS) purified from *A. muciniphila* HW07 activates the TLR4–IL-23–IL-22 immune axis and thereby ameliorates diet-induced obesity in mice (Sun et al., 2025). *A. muciniphila* can also regulate the secretion of gut hormones, such as enhancing the release of glucagon-like peptide-1 (GLP-1), thereby improving host glucose tolerance and insulin sensitivity (Yoon et al., 2021). Furthermore, *A. muciniphila* can affect the composition and function of the gut microbiota, promoting the production of SCFAs, which can regulate host energy metabolism and further improve obesity (Depommier et al., 2020). These research findings indicate that *A. muciniphila* has significant potential in regulating host energy metabolism and improving obesity and may become a novel probiotic preparation for the prevention and treatment of obesity and related metabolic diseases (Cani et al., 2022). However, *A. muciniphila* can have negative effects under certain disease conditions or specific circumstances. For example, one study found that *A. muciniphila* can modify IgA1 through deglycosylation, generating novel epitopes and exacerbating Immunoglobulin A nephropathy (IgAN), an autoimmune disease characterized by IgA-dominant immune deposits in the glomerular mesangium (Gleeson et al., 2024). Another study showed that in fiber – deficient diets, *A. muciniphila* degrades mucus and promotes type – 2 immune responses, worsening food allergies in mice (Parrish et al., 2022). A recent study also demonstrated that normally, ILC3s secrete IL – 22 to maintain a fucosylated barrier on intestinal cells, blocking harmful bacteria. When ILC3s are absent, galactosylation rises, allowing *A. muciniphila* to multiply and secrete succinate, altering the gut environment and increasing the risk of pathogen colonization and infection (Wang et al., 2025). Therefore, the characteristics of *Akkermansia*, an important gut probiotic, are not yet fully understood, and further research is needed to explore its potential applications.

In this study, 222 *Akkermansia* strains were isolated from the feces of healthy human donors. To reduce strain redundancy, one strain per donor was retained, with 26 strains selected for subsequent genomic analyses. These 26 isolates, combined with 97 additional strains retrieved from the NCBI database, were used to construct a comprehensive *Akkermansia* genomic dataset. Phylogenetic analysis of this dataset identified eight *Akkermansia* species, including two major clades (*A. muciniphila* and *Akkermansia massiliensis*) and two potential novel candidate species. Using an HFD-induced obesity model, we found that one selected strain, AKM Lab-01, significantly prevented HFD-induced weight gain. In an obesity treatment model (using pre-obese mice), AKM Lab-01 reduced the body weight of obese mice by 10%, lowered blood glucose and lipid levels, improved insulin resistance, and ameliorated indicators of metabolic syndrome following a 4-week intervention. Additionally, evaluation of probiotic properties showed that AKM Lab-01 exhibited strong probiotic potential, including tolerance to pH and bile salts, high auto-aggregation capacity, and antibiotic sensitivity. Moreover, both live, pasteurized, and powdered forms of AKM Lab-01 enhanced GLP-1 expression, with Amuc_1100 protein content at 350 ng/mg in viable preparations and 450 ng/mg in drug substance form; the strain also reduced serum LPS, TNF-α and IL-6 levels, which contributed to the improvement of obesity and associated metabolic disorders. A 90-day dietary exposure study was conducted to assess the safety of AKM Lab-01. The results revealed no adverse effects at doses up to 8 × 10^10^ TFU/day, in accordance with European Food Safety Authority (EFSA) guidelines. Collectively, these findings highlight the potential of AKM Lab-01 as a promising probiotic for the prevention and treatment of obesity and related metabolic diseases.

## 2 Materials and methods

### 2.1 Bacterial isolation and cultivation

The *Akkermansia spp*. strains isolated in this study originated from fecal samples of five healthy human subjects. Strain isolation refers to the method of Mushtaq, with some modifications (Mushtaq et al., 2021). Briefly, 1 g of fecal material was vortex-mixed with 9 mL of sterile physiological saline in an anaerobic chamber to prepare a 10^−1^ dilution. Serial ten-fold dilutions were then performed up to 10^−6^ dilution. A 100 μL aliquot of the 10^−6^ dilution was spread onto blood agar plates (Jiangmen Kailin P0903). After uniform spreading, plates were incubated in an inverted position at 37 °C under anaerobic conditions for 3–5 days to obtain isolated colonies. Single colonies were selected, and the near-full-length 16S rRNA gene was amplified using universal primers 27F and 1492R (Heuer et al., 1997). The resulting sequences were submitted to the NCBI Nucleotide BLAST database for analysis. Sequence comparisons confirmed that the isolates belonged to the genus *Akkermansia*. *A. muciniphila* AKM Lab-01 was isolated from a fecal sample donated by a healthy 56-year-old male volunteer in Dali, Yunnan, China, and has been deposited at Guangdong Microbial Culture Collection Center (GDMCC) under accession number GDMCC 63782. They were cultured in self-optimized MM01 liquid medium (peptone, 15 g; glucose, 20 g; yeast extract, 15 g; cysteine, 1 g; sodium acetate, 5 g; sodium citrate, 4 g; dipotassium phosphate, 2 g; magnesium sulfate, 0.1 g; manganese sulfate, 0.05 g; Tween 80, 1 g contained per liter; pH 6.3–6.5) and grown anaerobically under an atmosphere of 10% CO_2_, 10% H_2_ and 80% N_2_ at 37 °C for 48 h. All the reagents were vented in an anaerobic atmosphere for at least 24 h prior to use. Cultures were concentrated in deoxygenated PBS (containing 0.05% Cys-HCL) with 25% (v/v) glycerol under strictly controlled anaerobic conditions for use as a live bacterium. The *A. muciniphila* culture was pasteurized at 70 °C for 30 min, followed by immediate refrigeration at −80 °C until further use. In addition, pasteurized *A. muciniphila* was centrifuged and re-suspended in sterile pure water and then freeze-dried for 48 h in a vacuum freeze-dryer to obtain *A. muciniphila* drug substance.

### 2.2 Genome sequencing, assembly, and analysis

Bacterial genomic DNA was extracted from bacterial pellet samples using the ALFA-SEQ Fast Magnetic Bacteria DNA Kit (DC313-03, Findrop, China), following the manufacturer's instructions. DNA libraries were constructed using the VAHTS Universal Plus DNA Library Prep Kit for Illumina V4 (ND801, Vazyme, China) in accordance with the manufacturer's protocol. The whole genome shotgun sequencing was performed on the NextSeq 2000 platform. Reads were filtered with fastp (version 0.20.0), assembled with SPAdes (version 3.14.0), and annotated with PROKKA (version 1.14.5). The genome assembly accession number is JBQKMJ000000000, and the public repository URL address is https://www.ncbi.nlm.nih.gov/nuccore/JBQKMJ000000000. Orthofinder (version 2.5.5) was used for comparative analysis of 123 genomes, including strains from NCBI. The Clusters of Orthologous Groups (COGs) databases identified functional categories of gene families in *A. muciniphila* and *A. massiliensis*. A phylogenetic tree was constructed using IQ-TREE (version 2.2.2.7) with single-copy core genes aligned by MAFFT (version 7.520) and refined by Gblocks (version 0.91b; Minh et al., 2020). Given that Amuc_1409, Amuc_1100, and P9 proteins are well-recognized as core functional effectors mediating *Akkermansia*'s probiotic activities in metabolic regulation (e.g., intestinal barrier maintenance, inflammation modulation, and GLP-1 induction), we focused on these three proteins for homology analysis. Homology analyses were performed on Amuc_1409, Amuc_1100, and P9 proteins using blastp (version 2.14.0+) against the corresponding proteins in *A. muciniphila* ATCC BAA−835. Virulence factors were identified by Virulence Factor Database (VFDB).

### 2.3 Scanning electron microscopy (SEM)

Micrographs of *A. muciniphila* AKM Lab-01 were obtained using scanning electron microscopy (SEM). Bacterial cells were prefixed in 3 % (v/v) glutaraldehyde at 4 °C for 4–24 h, rinsed six times (20 min each) with 0.1 M phosphate-buffered saline (PBS, pH 7.0), and then dehydrated through an ethanol series: 30 % and 50 % (two changes, 10 min each), 70 % (overnight at 4 °C), 90 % (one change, 15 min) and 100 % (three changes, 15 min). Samples were critical-point-dried, sputter-coated with gold, and examined by SEM (Apreo 2S, Thermo Scientific).

### 2.4 Tolerance to pH and bile salts

The methods for assessing tolerance to pH and bile salts were referred to in a previous publication (Hassanzadazar et al., 2012). *A. muciniphila* AKM Lab-01 was initially inoculated into MM01 medium at 37 °C for 72 h. Subsequently, the activated *A. muciniphila* AKM Lab-01 was inoculated into MM01 medium adjusted to different pH levels (3 to 10) or containing various concentrations of bile salts (0 to 0.4%) and incubated for 48 h at 37 °C. After culture, the optical density at 600 nm was measured.

### 2.5 Evaluation of auto-aggregation

Auto-aggregation assessment was referred to the previous publication (Cozzolino et al., 2020), *A. muciniphila* AKM Lab-01 was cultured in MM01 medium and incubated at 37 °C for 48 h. Cell precipitates were obtained by centrifugation (4 °C; 6,000 rpm, 10 min), washed twice, and resuspended in phosphate-buffered saline (PBS, pH 7). The optical density was adjusted to 0.5 at 600 nm. The bacterial suspension was then incubated at 37 °C, and the optical density at 600 nm was measured every 30 min for up to 6 h. The auto-aggregation of AKM Lab-01 was calculated using the following formula (Cozzolino et al., 2020):


$$\begin{array}{l} \text{Auto-}aggregation\;\%\;=1-\left(\text{O}{\text{D}}_{\text{Final}}/OD_{0}\right)\;\times 100 \end{array}$$

where OD_0_ is the initial optical density at time 0, and OD_final_ is the initial optical density measured after 0.5, 1, 1.5, 2.5, 3.5, 4.5, 5, 5.5, and 6 h.

### 2.6 Minimal inhibitory concentration (MIC) of antibiotics

The method was referred to the previous publication (Francisco et al., 2023). *A. muciniphila* AKM Lab-01 was cultured in MM01 medium at 37 °C for 48 h. After incubation, cell precipitates were collected by centrifugation at 4 °C and 6,000 rpm for 10 min, washed twice, and resuspended in phosphate-buffered saline (PBS, pH 7). The optical density was adjusted to an OD_600_ of 1.0. A 100 μL aliquot of the bacterial suspension was then spread onto an MM01 agar plate. An antibiotic test strip (Liofilchem, Italy) was placed on the surface of the agar. The antibiotic diffuses radially into the agar, creating a concentration gradient that inhibits the growth of susceptible bacteria, forming a zone of inhibition. The size of this zone correlates with the antibiotic concentration. By measuring the intersection points of the inhibition zone with the test strip, the minimum inhibitory concentration (MIC) of the antibiotic against the specific bacterium was accurately determined (Table 1).

**Table 1:** Minimal inhibitory concentration (MIC) of antibiotics of AKM Lab-01.

**Antibiotic**	**MIC (mg/L)**
Ampicillin	4
Chloramphenicol	3
Clindamycin	0.13
Amoxicillin	0.05
Rifampicin	1
Imipenem	0.38
Penicillin	2
Cefquinoxime	1

### 2.7 Quantitative detection of Amuc_1100 protein by mass spectrometry

Quantitative detection of Amuc_1100 was performed by mass spectrometry with Multiple Reaction Monitoring (MRM) was referred to the previous publication with modifications (Zhang et al., 2011). Briefly, bacterial total protein was extracted using urea lysis buffer (24.0240 g urea, 0.6057 g Tris base in 40 mL), and the protein concentration was measured using the BCA assay (Bicinchoninic Acid Assay). Proteins were precipitated with acetone and redissolved in 8 M Urea. The proteins were reduced with DTT (Dithiothreitol, 10 mM) and alkylated with IAA (Iodoacetamide, 50 mM), then diluted to 1 M urea in the sample, enzymatically digested at 37 °C overnight at a 1:50 mass ratio (trypsin: protein) and finally desalted and lyophilized for mass spectrometry. The characteristic peptide of the Amuc_1100 protein was synthesized and subsequently used for analysis via triple quadrupole LC-MS/MS (Agilent 1290-6470B) to establish a quantitative standard curve for Amuc_1100. Based on this standard curve, the signal intensity of the characteristic peptide in the samples was quantified by mass spectrometry, allowing the final determination of the Amuc_1100 protein content in the samples.

### 2.8 Experimental animals

Five to six weeks old male C57BL/6J mice (GemPharmatech Inc.) were maintained in a specific pathogen free (SPF) environment. All animal experiments were carried out according to protocols approved by the Institutional Animal Care and Use Committee of Moon Biotech Co., Ltd, in compliance with the Guide for the Care and Use of Laboratory Animals. All mice were cared for under controlled temperature and humidity (24 ± 2 °C, 50% ± 10%) with a 12 h light/dark cycle. Animals exhibiting abnormal behavior or physiological conditions were excluded prior to group assignment. All mice were randomly assigned to experimental groups by body weight to ensure balanced distribution across groups. Experimental groups were identified exclusively by alphanumeric codes, and the experimenters were unaware of treatment group identities.

For (i) **Experiment 1** (IACUC Number: IACUC202407-4O, a prevention model): Forty-two male C57BL/6J mice were randomly divided into 7 groups after a 1-week acclimation period: one normal chow diet (NCD) group (normal diet: 3.8 kcal/g, with 20% of energy derived from protein, 10% from fat, and 70% from carbohydrate; Diet D12450B; Research Diets, Inc., New Brunswick, New Jersey, USA) and six high-fat diet (HFD)-fed groups [HFD: 5.2 kcal/g, with 20% of energy derived from protein, 60% from fat, and 20% from carbohydrate (Diet D12492; Research Diets, Inc., New Brunswick, New Jersey, USA)] (*n* = 6 mice per group). All diets were administered for 8 weeks. Mice in the *A. muciniphila* S1–S5 groups (AKK groups) received daily oral gavage of the corresponding *A. muciniphila* strains [10^9^ colony-forming units (CFU), used for live bacteria counting, in 200 μL PBS], while those in the NCD and HFD groups received 200 μL PBS via oral gavage from Day 1. According to the experimental protocol, body weight and food intake was measured twice weekly. On Day 56, mice were euthanized by cervical dislocation following anesthesia induction with 4–5% isoflurane (R510-22-10, Jinan Ante Biochemical Pharmaceutical Co., Ltd.) in oxygen (200–400 mL/min) delivered via an inhalation anesthesia system (CL-1000-S4, Shanghai Yuyan Instruments Co., Ltd.) within an induction chamber.

For (ii) **Experiment 2** (IACUC Number: IACUC202407-14O, a treatment model): After 1 week of acclimatization, 20 mice were fed HFD (D12492; Research Diets, inc., New Brunswick, New jersey) for 10 weeks to build an obesity model. Subsequently, 12 mice weighing 38–46 g were selected and randomly divided into two groups: HFD-Control group and viable AKM Lab-01 group. Obese mice in these groups received daily oral gavage (0.2 mL per animal) for 28 days: PBS for the HFD-Control group (*n* = 6) and viable AKM Lab-01 (10^9^ CFU) for the treatment group (*n* = 6) from week 10. According to experimental procedure, body weight and 24 h food intake was measured weekly or twice weekly. One week before the experiment's endpoint, glucose tolerance tests were conducted as follows: After a 12-h fasting period, the mice were weighed and administered a glucose solution at a dose of 2 g/kg body weight via oral gavage. Blood samples were collected from the tail tip at 0, 15, 30, 60, 90, and 120 min to measure serum glucose levels. Glucose concentrations were determined using a glucometer with glucose test strips. On Day 28, all mice underwent an overnight fast prior to terminal procedures. An inhalation anesthesia apparatus (CL-1000-S4, Shanghai Yuyan Instruments Co., Ltd.) was utilized for mouse sedation. Within the induction chamber, animals received 4–5% isoflurane (R510-22-10, Jinan Ante Biochemical Pharmaceutical Co., Ltd.) in oxygen (flow rate: 200–400 mL/min) until loss of righting reflex was observed. The mice were then positioned in a nose cone for maintenance of surgical anesthesia using 1.5–2.5% isoflurane. Orbital venous plexus blood collection was carried out under deep terminal anesthesia, immediately followed by euthanasia via cervical dislocation. Adipose depots (epididymal fat, perirenal fat, mesenteric fat and Inguinal fat) and liver were precisely dissected and weighed. Epididymal fat, liver and colon tissues were fixed in paraformaldehyde or Karnoy's solution and subjected to histological staining and analysis. Serum chemistry parameters (TCHO, TG, LDL, HDL, ALT, AST, BUN, and CRE) were tested by Wuhan Servicebio Technology Co., Ltd. using automatic biochemical analyzer.

For (iii) **Experiment 3** (IACUC Number: IACUC202408-7O, a treatment model): After 1 week of acclimatization, 40 C57BL/6J male mice were randomly divided into 5 groups according to body weight (8 mice/group, 4 mice per cage). The NCD group were fed a normal control diet (D12450B; Research Diets, inc., New Brunswick, New jersey). The remaining four groups were fed HFD (D12492; Research Diets, inc., New Brunswick, New jersey). After 4 weeks on HFD, the mice had reached mild obesity (body weight 28–34 g) and were stratified by weight into four groups (*n* = 8 each): HFD-Control, viable AKM Lab-01 (AKM Lab-01-L, 10^10^ CFU per animal), pasteurized AKM Lab-01 (AKM Lab-01-P, 10^10^ CFU per animal), and pasteurized AKM Lab-01 drug substance (AKM Lab-01-D, 10^10^ CFU per animal). From week 5 onward the NCD-Control and HFD-Control groups received 0.2 mL PBS daily by oral gavage for 56 days, while the three treatment groups received 0.2 mL of the corresponding bacterial preparation daily for the same period. According to the experimental procedure, body weight and food intake was measured twice weekly. On Day 56, after an overnight fast, mice were anesthetized with isoflurane (4–5% induction, 1.5–2.5% maintenance). Orbital venous plexus blood was collected under deep anesthesia before euthanasia by cervical dislocation. Tissues and fecal samples were then harvested from all animals for subsequent analyses.

For (iii) **Experiment 4** (IACUC Number: IACUC202507-4O, a treatment model): After a 1-week acclimatization, thirty male C57BL/6J mice (6 weeks old) were fed a high-fat diet (HFD; D12492, Research Diets, New Brunswick, NJ, USA) for 10 weeks to induce obesity. Eighteen animals that reached 36–44 g were then selected and randomly assigned to three groups (*n* = 6 each): The HFD-Control group received 0.2 mL PBS daily, while the AKM Lab-01 and BAA-835 groups received 1 × 10^9^ CFU of the respective live strain in 0.2 mL PBS daily; all gavages were given once daily for 28 consecutive days from week 10. Body weight and food intake was measured twice weekly. On Day 28, after overnight fasting, mice were anesthetized with isoflurane (4–5% induction, 1.5–2.5% maintenance) for orbital blood collection under deep anesthesia, followed by cervical dislocation euthanasia and tissue harvesting.

### 2.9 Histological staining and analysis

Liver and adipose tissue samples were fixed in paraformaldehyde, stained with hematoxylin and eosin (H&E), and examined for pathology by Wuhan Servicebio Technology Co., Ltd. The tissue was photographed at 200 × magnification. The severity of non-alcoholic fatty liver disease (NAFLD) was assessed using inflammation, steatosis, and ballooning scores, as well as the NAFLD activity score (NAS).

The thickness and integrity of the colonic mucus layer were evaluated using Alcian Blue staining. Colon tissues were fixed in Karnoy's solution and stained with Alcian Blue, which selectively binds to acidic mucins, resulting in blue staining. The tissues were photographed at 200 × magnification. The mucus layer thickness was measured under a light microscope to assess the structural integrity of the colonic mucus.

### 2.10 Detection of blood GLP-1

Two hundred fifty microliter blood was quickly mixed with 0.1 nmol/L Dipeptidylpeptidase IV Inhibitor I (Sigma, 416200), 7.5 μL 0.5 M EDTA, and 2.5 μL Protease Inhibitor Cocktail (Sigma, P8340)). Plasma was then isolated by centrifugation and stored at −80 °C until subsequent hormone analysis. Total GLP-1 was measured using Glucagon-Like Peptide-1 (Active) ELISA kit (Merck, EGLP-35K) according to the provided protocols.

### 2.11 Metagenomic analysis

Bacterial genomic DNA was extracted by Guangdong Magigene Biotechnology Co., Ltd. (Guangzhou, China) using commercial kits. DNA integrity and purity were monitored on 1% agarose gels. DNA concentration and purity were measured using Qubit 3.0 (Thermo Fisher Scientific, Waltham, USA) and Nanodrop One (Thermo Fisher Scientific, Waltham, USA). Sequencing libraries were generated using ALFA-SEQ DNA Library Prep Kit. The library quality was assessed on the Qubit 4.0 Fluorometer (Life Technologies, Grand Island, NY, USA) and Qsep400 High-Throughput Nucleic Acid Protein Analysis system (Houze Biological Technology Co., Hangzhou, China). At last, the library was sequenced on an Illumina NovaSeq 6000 platform and 150 bp paired-end reads were generated. Raw sequencing reads were quality-filtered using fastp (version 0.20.0). Kraken2 (version 2.1.3) and Bracken (version 2.9) were used to remove host reads and to generate the relative abundance in each sample. Subsequent alpha diversity analysis, PCoA and non-parametric test were performed on R scripts.

### 2.12 ELISA assay

Serum samples collected from the mouse study (Experiment 3) were used to quantify the concentrations of lipopolysaccharides (LPS), tumor necrosis factor α (TNF-α), and interleukin-6 (IL-6) via enzyme-linked immunosorbent assay (ELISA), following the manufacturers' instructions with specific kits: LPS was detected using the Mouse Lipopolysaccharides (LPS) ELISA Kit (Cusabio, Catalog No.: CSB-E13066m), TNF-α using the Mouse Tumor Necrosis Factor α (TNF-α) ELISA Kit (Cusabio, Catalog No.: CSB-E04741m), and IL-6 using the Mouse IL-6 ELISA Kit (MULTI SCIENCES, Catalog No.: EK206). Serum samples were diluted to the recommended concentrations, absorbance was measured at the specified wavelengths, and target molecule concentrations were calculated via standard curve fitting; each sample was assayed in triplicate to ensure data reliability.

### 2.13 90-day subchronic toxicity study

To evaluate the potential systemic toxicity of AKM Lab-01 drug substance (freeze-dried pasteurized *A. muciniphila* Lab-01), a 90-day subchronic oral toxicity study was conducted *in vivo* according to the OECD guidelines (IACUC Number: IACUC202408-5AT). Forty 6- to 7-week-old BALB/c mice (20 males and 20 females) were obtained from GemPharmatech Inc. and housed under controlled conditions (temperature 24 ± 2 °C, humidity 50% ± 10%, 12-h light/dark cycle). After a 1-week acclimatization period, the mice were randomly divided into four groups: Group 1 received saline only; Groups 2, 3, and 4 received AKM Lab-01 drug substance at dosages of 8.8 × 10^10^, 8.8 × 10^9^, and 8.8 × 10^8^ TFU/mouse per day, respectively. The suspensions were administered orally and daily for 90 days. All the mice were fed standard maintenance chow diet (3.8 kcal/g, with 21.5% of energy derived from protein, 11.1% from fat and 67.4% from carbohydrate; Diet XTI01WC-009, Jiangsu Province Collaborative Pharmaceutical Biotechnology Engineering Co., Ltd., China) during the whole study. Clinical signs were recorded daily, and comprehensive clinical observations were performed weekly. Body weight, food intake, and water consumption were monitored weekly. After 90 days, following an overnight fast, mice received isoflurane anesthesia (induction: 4–5%; maintenance: 1.5–2.5%). Orbital venous plexus blood was collected under deep terminal anesthesia before euthanasia by cervical dislocation. Tissue specimens were subsequently harvested from all animals. Blood samples were analyzed for hematology, coagulation, and clinical chemistry, while tissues were examined for macroscopic changes, organ weights, and histopathology.

### 2.14 Statistical analysis

Statistical analysis was performed using GraphPad Prism (version 10.2.3), and data are presented as the mean ± standard deviation (Mean ± SD) unless otherwise indicated. Raw data from *in-vitro* assays were pre-processed with Cytation5 software (version 3.11.19) prior to analysis. Normality was assessed with the Shapiro–Wilk test, and homogeneity of variances with Levene's test; outliers were identified and excluded using the ROUT method (*Q* = 1%). For multiple-group comparisons among more than three groups, one-way ANOVA with Dunnett's multiple comparison test was applied. For all pairwise comparisons following ANOVA, Tukey's multiple comparisons test was applied. When analyzing two independent variables, two-way ANOVA with Dunnett's *post hoc* test was used. For two-group comparisons, a two-tailed unpaired t-test was used when data were normally distributed and variances were equal; otherwise, the Mann–Whitney *U* test was applied. All graphical representations were generated using GraphPad Prism (version 10.2.3). Significance levels are indicated as: ns, not significant; ^*^p < 0.05; ^**^p < 0.01; ^**^p < 0.001; ^****^p < 0.0001.

## 3 Results

### 3.1 Phylogenetic analysis, genomic and functional comparation of *A. muciniphila* AKM Lab-01 and closely related species

**Figure 1:**
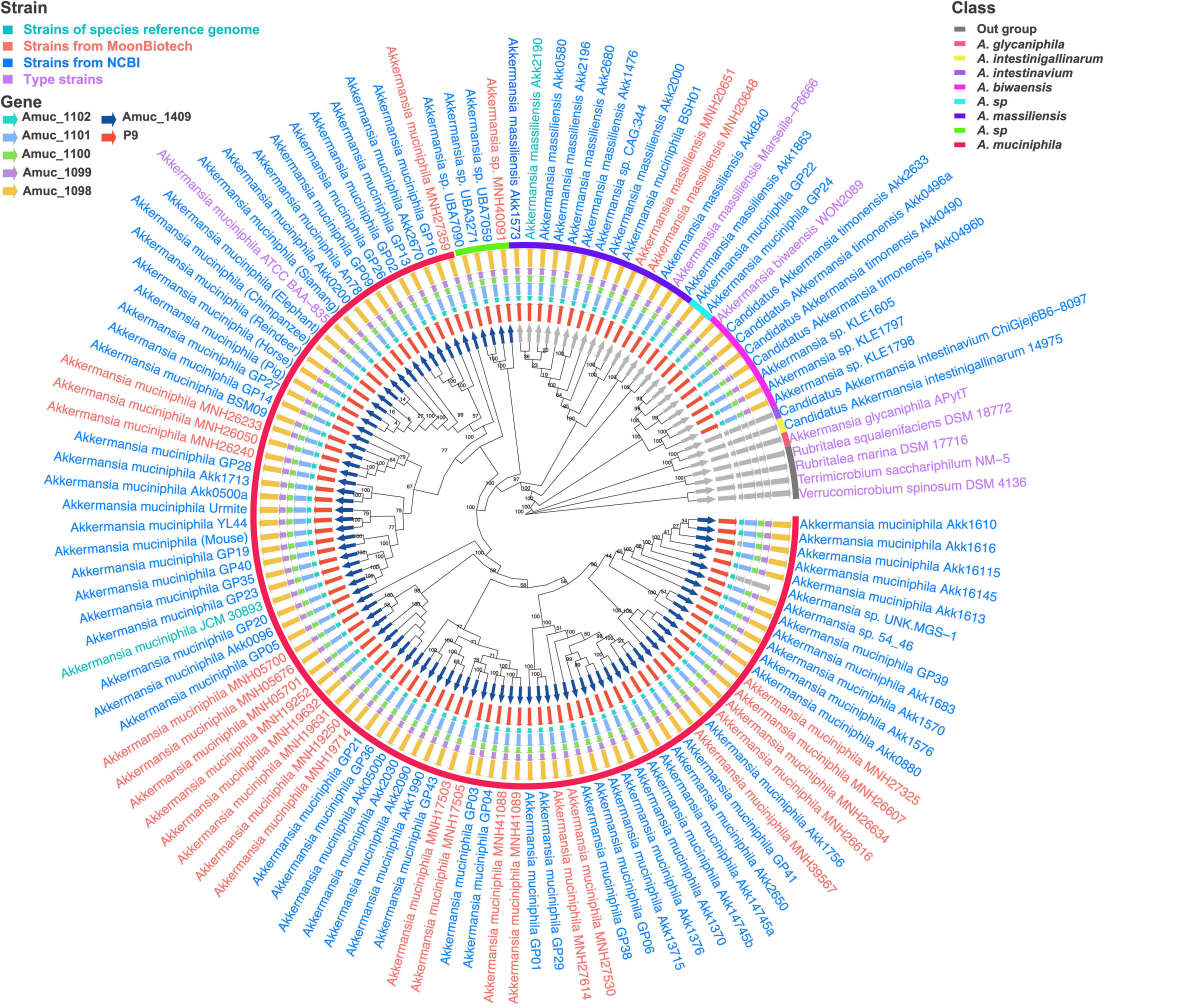
Phylogenetic relationship of *Akkermansia* species. Phylogenetic tree was constructed using IQ-TREE (version 2.2.2.7) with default parameters under the maximum likelihood (ML) criterion, based on 338 single-copy core genes. The core gene sequences were aligned by MAFFT (version 7.520) and refined by Gblocks (version 0.91b); ultrafast bootstrap approximation (UFBoot) with 1,000 replicates was used to assess branch support. Numbers represent bootstrap values. *Verrucomicrobium spinosum* DSM 4136, *Rubritalea marina* DSM 17716, *Terrimicrobium sacchariphilum* NM-5, and *Rubritalea squalenifaciens* DSM 18772 were used as out-groups. Homology analyses of Amuc_1409, Amuc_1100, and P9 proteins were performed using BLASTp (version 2.14.0+) against the corresponding proteins in *A. muciniphila* ATCC BAA−835.

In our study, we isolated 222 strains of *Akkermansia* from healthy human feces. To minimize strain redundancy, we retained only one strain isolated from each donor's fecal sample and selected 26 strains for whole-genome sequencing. These 26 strains, together with 97 additional strains obtained from public databases, were used to form a comprehensive genomic dataset of *Akkermansia* strains, designated as Supplementary Dataset S1. Firstly, a phylogenetic tree was constructed using IQ-TREE based on 338 single-copy core genes (Supplementary Dataset S4). *Verrucomicrobium spinosum* DSM 4136, *Rubritalea marina* DSM 17716, *Terrimicrobium sacchariphilum* NM-5, and *Rubritalea squalenifaciens* DSM 18772 were selected as out-groups. To ensure the accuracy of our pan-genome analysis, strains were identified based on the following criteria: they must be in the same branch of the phylogenetic tree, exhibit ≥95% Average Nucleotide Identity (ANI) with the type strain of the species (if available), show ≥95% ANI with other strains within the same species, and have < 95% ANI with strains of other species. Based on these criteria, we labeled strains in the same branch as the type strains of *A. muciniphila* (*n* = 88) and *A. massiliensis* (*n* = 14) for further analysis (Figure 1). We found that strains such as *Akkermansia* sp. CAG:344 and *Akkermansia muciniphila* BSH01 were in the same branch as the type strain of *Akkermansia massiliensis*, suggesting potential inaccuracies in previous species identification (Guo et al., 2017; Xing et al., 2019).

Additionally, two potential novel species were identified. The first potential novel species, including *Akkermansia* sp. MNH40091, *Akkermansia* sp. UBA7059, *Akkermansia* sp. UBA3271, and *Akkermansia* sp. UBA7090, was closely related to *A. muciniphila*. The second potential novel species, including *Akkermansia muciniphila* GP22 and *Akkermansia muciniphila* GP24, was in an outer branch of the large clade containing *A. muciniphila, A. massiliensis*, and the first potential novel species (Figure 1). Moreover, strains previously identified as *Candidatus Akkermansia timonensis* (including *Candidatus Akkermansia timonensis* Akk2633, *Candidatus Akkermansia timonensis* Akk0496a, *Candidatus Akkermansia timonensis* Akk0490, *Candidatus Akkermansia timonensis* Akk0496b) and *Akkermansia* sp. (including *Akkermansia* sp. KLE1605, *Akkermansia* sp. KLE1797, *Akkermansia* sp. KLE1798) were reclassified as *Akkermansia biwaensis* (Figure 1). In total, eight *Akkermansia* species were included in our study: *A. muciniphila, Akkermansia massiliensis*, the potential novel species closely related to *A. muciniphila*, the potential novel species in the outer branch, *Akkermansia biwaensis, Candidatus Akkermansia intestinavium, Candidatus Akkermansia intestinigallinarum*, and *Akkermansia glycaniphila*.

Subsequently, we conducted a pan-genome analysis. For *A. muciniphila*, the analysis revealed a total of 4,167 gene families, with 1,448 core gene families shared among the 88 strains (Supplementary Dataset S2). The rarefaction curve showed that the increase in both total and core gene families slowed down as new strains were added, indicating that our sample size for *A. muciniphila* is representative (Supplementary Figure S1A). In contrast, *A. massiliensis* had a total of 2,952 gene families, with 1,817 core gene families among the 14 strains (Supplementary Dataset S2). Given that *A. massiliensis* is a newly discovered species, its strain number is significantly lower than that of *A. muciniphila*. The instability in the rarefaction curves for both total and core gene families indicates that the sample size for *A. massiliensis* is insufficient (Supplementary Figure S1B).

**Figure 2:**
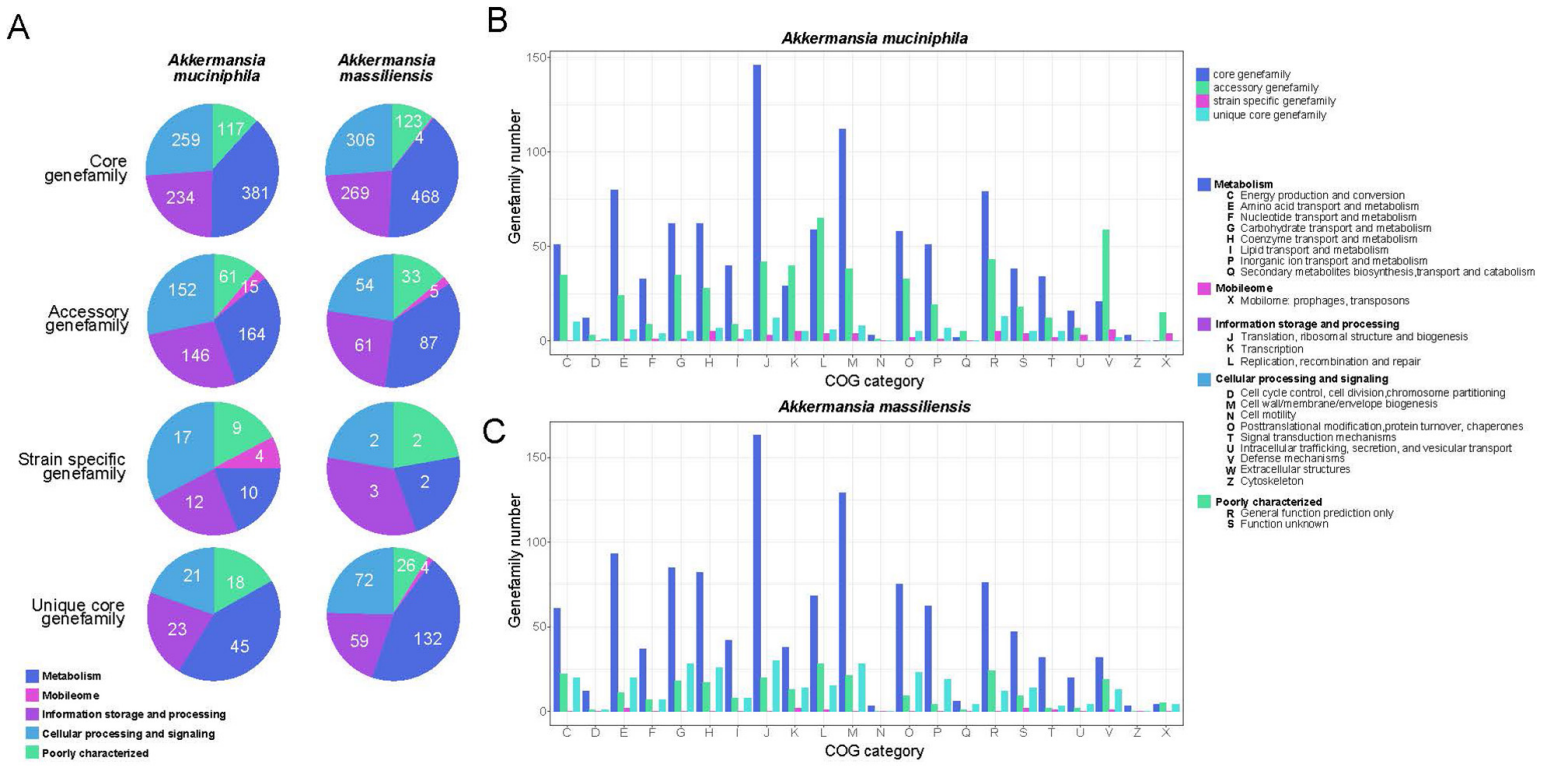
Pan-genome analyses of *Akkermansia muciniphil*a and *Akkermansia massiliensis*. **(A)** Proportion of five classes of functional categories in core, accessory, strain-specific and species unique core genefamilies of *Akkermansia muciniphila* and *Akkermansia massiliensis*. **(B, C)** Functional categories in core, accessory, strain-specific and species unique core genefamilies of *Akkermansia muciniphila* **(B)** and *Akkermansia massiliensi*s **(C)**.

After obtaining core and strain-specific gene sequences, we compared their functional categories using the Clusters of Orthologous Groups (COGs) database (Tatusov et al., 2003). In *A. muciniphila*, COG annotation covered 75.7% (1096/1448) of core gene families, 27.4% (575/2099) of accessory gene families, and 9.4% (58/620) of strain-specific gene families. In contrast, in *A. massiliensis*, COG annotation covered 71.1% (1292/1817) of core gene families, 26.3% (256/975) of accessory gene families, and 5.6% (9/160) of strain-specific gene families. When comparing species-unique core gene families, *A. muciniphila* had 64.7% (121/187) annotated, while *A. massiliensis* had only 57.4% (319/556) annotated (Figure 2A, Supplementary Dataset S2). The lower annotation rates, especially for accessory and strain-specific gene families, suggest that the functions of many strains in these two species remain poorly studied. The COG annotation results for *A. muciniphila* core gene families were consistent with previous research at the functional class level (Xing et al., 2019), while *A. massiliensis* core gene families showed a similar functional class composition. The species-unique core gene families between *A. muciniphila* and *A. massiliensis* were primarily annotated as metabolism, cellular processing and signaling, and information storage and processing (Figure 2A). Detailed annotations showed that the three most abundant functions among core gene families annotated as metabolism were amino acid transport and metabolism, carbohydrate transport and metabolism, and coenzyme transport and metabolism (Figures 2B, C). Notably, *A. massiliensis* had more core gene families annotated in these categories than *A. muciniphila*.

Homology analyses of key proteins—Amuc_1409, Amuc_1100, and P9—revealed that Amuc_1409 is conserved in *A. muciniphila* and closely related new species. Meanwhile, Amuc_1100 and P9 are present in most strains of the *Akkermansia* genus, except for strains such as *Candidatus Akkermansia intestinavium* ChiGjej6B6-8097, *Candidatus Akkermansia intestinigallinarum* 14975, and *Akkermansia glycaniphila* APytT (Figure 1). Previous studies have shown that Amuc_1409 promotes gut health by enhancing intestinal stem cell proliferation and regeneration (Kang et al., 2024). Therefore, *A. muciniphila* and closely related new species are likely to share similar probiotic functions, such as promoting intestinal epithelial development and improving barrier integrity. Amuc_1100 strengthens epithelial tight junctions and reduces body weight and fat mass through TLR2 activation (Plovier et al., 2017; Wang et al., 2020, 2021). Additionally, this protein triggers a range of anti- and pro-inflammatory cytokines, thereby preventing obesity, insulin resistance, and inflammation in visceral adipose tissue (Si et al., 2022). Moreover, P9 induces the secretion of glucagon-like peptide-1 and activates brown adipose tissue thermogenesis (Yoon et al., 2021). These findings suggest that most strains of the *Akkermansia* genus possess probiotic functions associated with weight loss.

### 3.2 *Akkermansia muciniphila* AKM Lab-01 improve diet-induced obesity and related metabolic disorders including lipid metabolism

**Figure 3:**
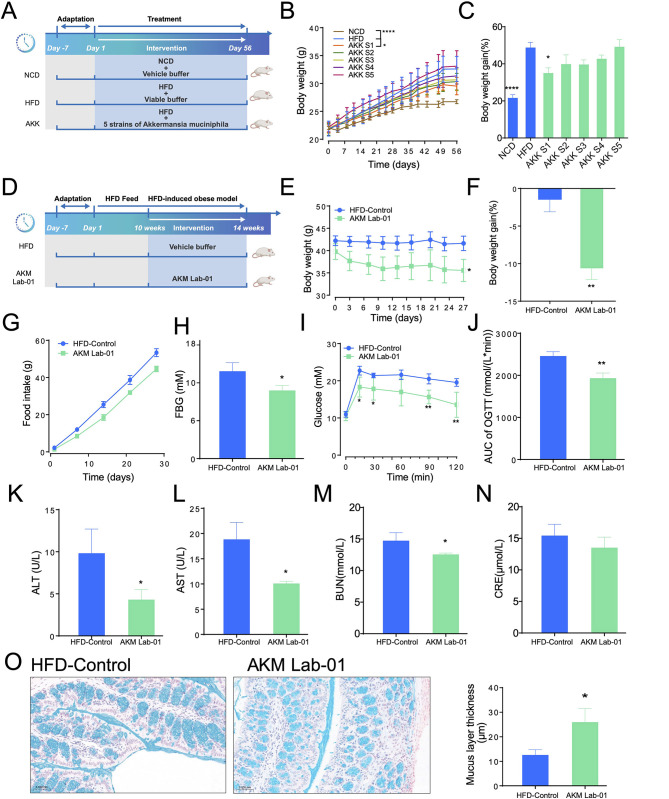
*Akkermansia muciniphila* AKM Lab-01 improve Diet-Induced Obesity and Related Metabolic Disorders. **(A–C)** Effects of five *Akkermansia* strains on HFD-induced obese mice. Male C57B6/J mice, aged 5–6 weeks, were divided into seven groups following a one-week acclimation period. The NCD group was fed a normal diet, while the other six groups (HFD and *A. muciniphila* S1–S5) were given an HFD. The dietary regimens lasted for 8 weeks. The *A. muciniphila* S1–S5 groups received daily oral gavage of *A. muciniphila* strains (10^9^ CFU in 200 μL PBS, *n* = 6 mice per group), whereas the NCD and HFD groups were administered 200 μL PBS (*n* = 6 mice per group). **(A)** Schematic diagram. **(B)** Curve graph of body weight. **(C)** Body weight gain (%) at on Day56. **(D–O)** *Akkermansia muciniphila* AKM Lab-01 alleviates HFD-induced obesity. Five to six weeks old male C57B6/J were fed a HFD for 10 weeks to establish an obesity model. Following this, the obese mice received either PBS (HFD-Control group, *n* = 6) or viable AKM Lab-01 (*n* = 6, 10^9^ CFU) via oral gavage for 28 days. **(D)** Schematic diagram. **(E)** Curve graph of body weight. **(F)** Body weight gain (%) at the endpoint. **(G)** Curve graph of cumulative food intake during intervention. **(H)** Fasting blood glucose. **(I)** Curve graph of OGTT. **(J)** Area under curve of OGTT. **(K)** Serum levels of ALT. **(L)** Serum levels of AST, **(M)** Serum levels of BUN. **(N)** Serum levels of CRE. **(O)** Representative alcian blue staining photographs of colon tissue at 200 × magnification. Data are presented as Mean±SD. Statistical analysis was performed by two-way ANOVA for the line chart **(B, E, I)** or one-way ANOVA for the bar chart combined with Dunnett's multiple comparisons test **(C)** and two-tail unpaired Student's *t* test for comparison between HFD-Control and AKM Lab-01 **(F, H, J–O)**, *p < 0.05, **p < 0.01, ****p < 0.0001.

To identify the optimal *Akkermansia* strain for preventing obesity and improving related metabolic disorders, we conducted an exploratory, product-oriented screening of 26 isolates in our strain library. First, genome-based prediction of virulence factors and core-genome COG annotation were performed; all isolates carried similarly low burdens of putative virulence genes, and functional profiles for amino-acid, carbohydrate and coenzyme transport/metabolism were highly similar across the set (Supplementary Dataset S3). Consequently, these genomic criteria served as qualitative references rather than exclusion filters. Next, cultivation performance was quantified by OD600 after 72 h in MM01 medium (Supplementary Figure S2); the five isolates exhibiting the highest biomass yield (S1 > S2 ≈ S3 ≈ S4 ≈ S5) were selected for *in-vivo* testing. These five isolates consist of four *A. muciniphila* strains—representing the most prevalent species in healthy human gut samples—and one *A. massiliensis* strain, which is the second-most abundant species. COG analysis revealed that *A. massiliensis* harbors a greater number of species-specific core gene families across several functional sub-categories than *A. muciniphila*, indicating a potentially broader substrate-utilization capacity (Figure 2A, Supplementary Dataset S2 and Supplementary Dataset S3). Consequently, these five *Akkermansia* strains were selected for efficacy evaluation in HFD-induced obese mice model with supplementation concurrently with the high-fat diet (Figure 3A). After an 8-week intervention, *A. muciniphila* S1 exhibited a substantial inhibitory effect on weight gain, significantly outperforming the HFD-Control group (Figures 3B, C). *A. muciniphila* S2, S3, S4, and S5 also demonstrated trends toward weight gain prevention, although these effects did not reach statistical significance (Figures 3B, C). Given its superior efficacy, *A. muciniphila* S1 was selected for further development and was renamed AKM Lab-01.

To assess the effects of AKM Lab-01 on diet-induced obesity and associated metabolic disorders, we established an obesity model in mice by feeding them HFD for 10 weeks, followed by a 4-week intervention with AKM Lab-01 (Figure 3D). After the intervention, we evaluated several key indicators, including body weight, food intake, blood glucose levels, liver and kidney function, and gut barrier integrity. The results demonstrated that AKM Lab-01 significantly reduced body weight gain (Figures 3E, F) in HFD-induced obese mice compared to the HFD-Control group. Additionally, AKM Lab-01 treatment led to a significant decrease in food intake (Figure 3G), fasting blood glucose levels (Figure 3H), and area under the curve (AUC) during an oral glucose tolerance test (OGTT; Figures 3I, J). Furthermore, AKM Lab-01 significantly improved liver function, as evidenced by reduced levels of alanine aminotransferase (ALT, Figure 3K) and aspartate aminotransferase (AST, Figure 3L). It also enhanced kidney function, with significant reductions in blood urea nitrogen (BUN, Figure 3M) and creatinine (CRE, Figure 3N). Moreover, AKM Lab-01 increased the thickness of the colonic mucus layer (Figure 3O), indicating improved gut barrier function. Collectively, these findings suggest that AKM Lab-01 can effectively mitigate diet-induced obesity and associated metabolic disorders by modulating multiple physiological pathways, including energy balance, glucose metabolism, liver and kidney function, and gut barrier integrity.

**Figure 4:**
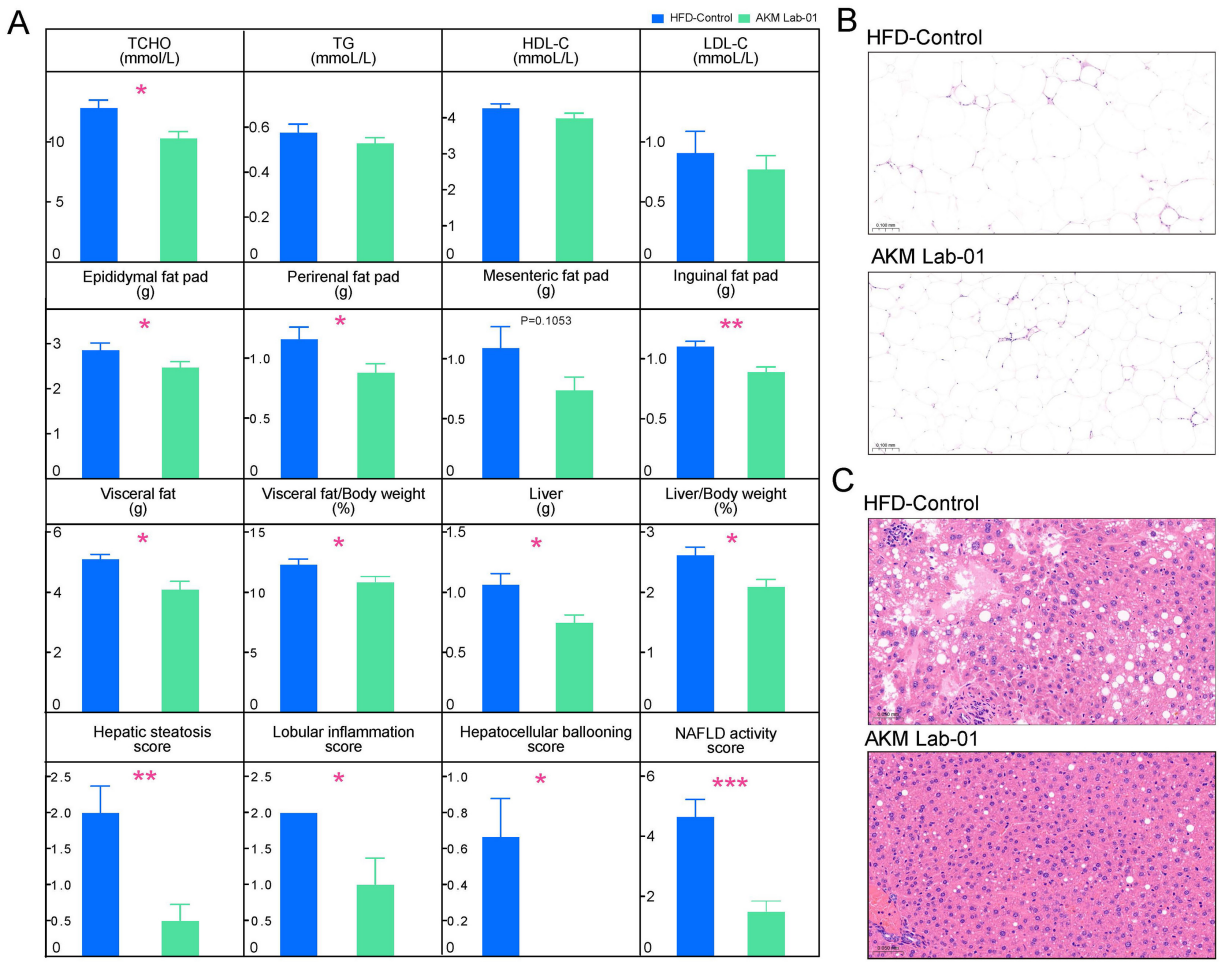
AKM Lab-01 improve lipid metabolism in HFD-induced obese mice. **(A)** Serum levels of TCHO, TG, HDL-C and LDL-C. Weight of epididymal fat pad, perirenal fat pad, mesenteric fat pad, inguinal fat pad, visceral fat, visceral fat/body weight (%), liver and liver/body weight (%), Hepatic steatosis score, Lobular inflammation score, Hepatocellular ballooning score, NAFLD activity score. **(B)** HE stained sections of epididymal fat. **(C)** HE stained sections of liver at 200× magnification. Data are presented as Mean ± SD. Statistical analysis was performed by two-tail unpaired Student' s *t* test for comparison between HFD-Control and AKM Lab-01 (*n* = 6 mice per group). *ns*, not significant, *p < 0.05, **p < 0.01, ***p < 0.001.

To further elucidate the effects of AKM Lab-01 on lipid metabolism in HFD-induced obese mice, we examined its impact on serum lipid profiles, including total cholesterol (TCHO), triglycerides (TG), high-density lipoprotein cholesterol (HDL-C), and low-density lipoprotein cholesterol (LDL-C) during the 4-week intervention period (Figure 4A). AKM Lab-01 significantly reduced serum TCHO levels compared to the HFD-Control group (Figure 4A).

In addition to its effects on serum lipids, AKM Lab-01 also exerted significant effects on adipose tissue. It markedly decreased the weights of epididymal fat, perirenal fat, mesenteric fat, and inguinal fat, as well as the proportion of visceral fat to body weight (Figure 4A). Histopathological analysis of adipose tissue revealed that AKM Lab-01 reduced adipocyte size, indicating a reduction in adipose tissue hypertrophy (Figure 4B). Regarding liver health, AKM Lab-01 significantly decreased liver weight and the proportion of liver weight to body weight (Figure 4A). Histopathological analysis of liver tissue showed that AKM Lab-01 reduced hepatic lipid accumulation, lobular inflammation, hepatocellular ballooning, and the non-alcoholic fatty liver disease (NAFLD) activity score compared to the HFD-Control group (Figure 4C). Collectively, these findings demonstrate that AKM Lab-01 can significantly improve lipid metabolism and ameliorate adipose tissue and liver pathology in HFD-induced obese mice, suggesting its potential as a therapeutic agent for obesity-related metabolic disorders. To benchmark the anti-obesity efficacy of AKM Lab-01, we compared it with the widely used reference strain, live *A. muciniphila* ATCC BAA-835. After 10 weeks of HFD induction, obese mice were treated with 1 × 10^9^ CFU of AKM Lab-01 or BAA-835 daily for 28 days. Both strains significantly reduced cumulative weight gain compared to HFD-Control, with no significant difference between them, indicating that AKM Lab-01 is at least as effective as the standard strain in this obesity model (Supplementary Figure S5).

### 3.3 Taxonomic status and genomic distinctiveness of *A. muciniphila* AKM Lab-01

To clarify *A. muciniphila* AKM Lab-01′s taxonomic status and genomic distinctiveness with reference *Akkermansia* strains, we performed the following analyses: First, we aligned the 16S rRNA sequence of AKM Lab-01 with that of reference *Akkermansia* strains. Phylogenetic analysis of the 16S rRNA gene confirmed that AKM Lab-01 clusters within the same clade as other validated *A. muciniphila* strains (Supplementary Figure S3A). Furthermore, AKM Lab-01 shared >99% ANI with *A. muciniphila* BAA-835 (Supplementary Figure S3B), confirming AKM Lab-01′s assignment to the species *A. muciniphila*. Subsequently, we performed comparative genomic analysis through COG annotation of AKM Lab-01, BAA-835, and three additional *A. muciniphila* strains examined in this study. This analysis revealed a relative enrichment of genes assigned to COG category P (Inorganic ion transport and metabolism) in AKM Lab-01 (Supplementary Figure S3C). This distinctive genomic signature may indicate unique functional attributes potentially contributing to its probiotic characteristics, though the underlying mechanism requires further study.

### 3.4 General probiotic properties of *A. muciniphila* AKM Lab-01

**Figure 5:**
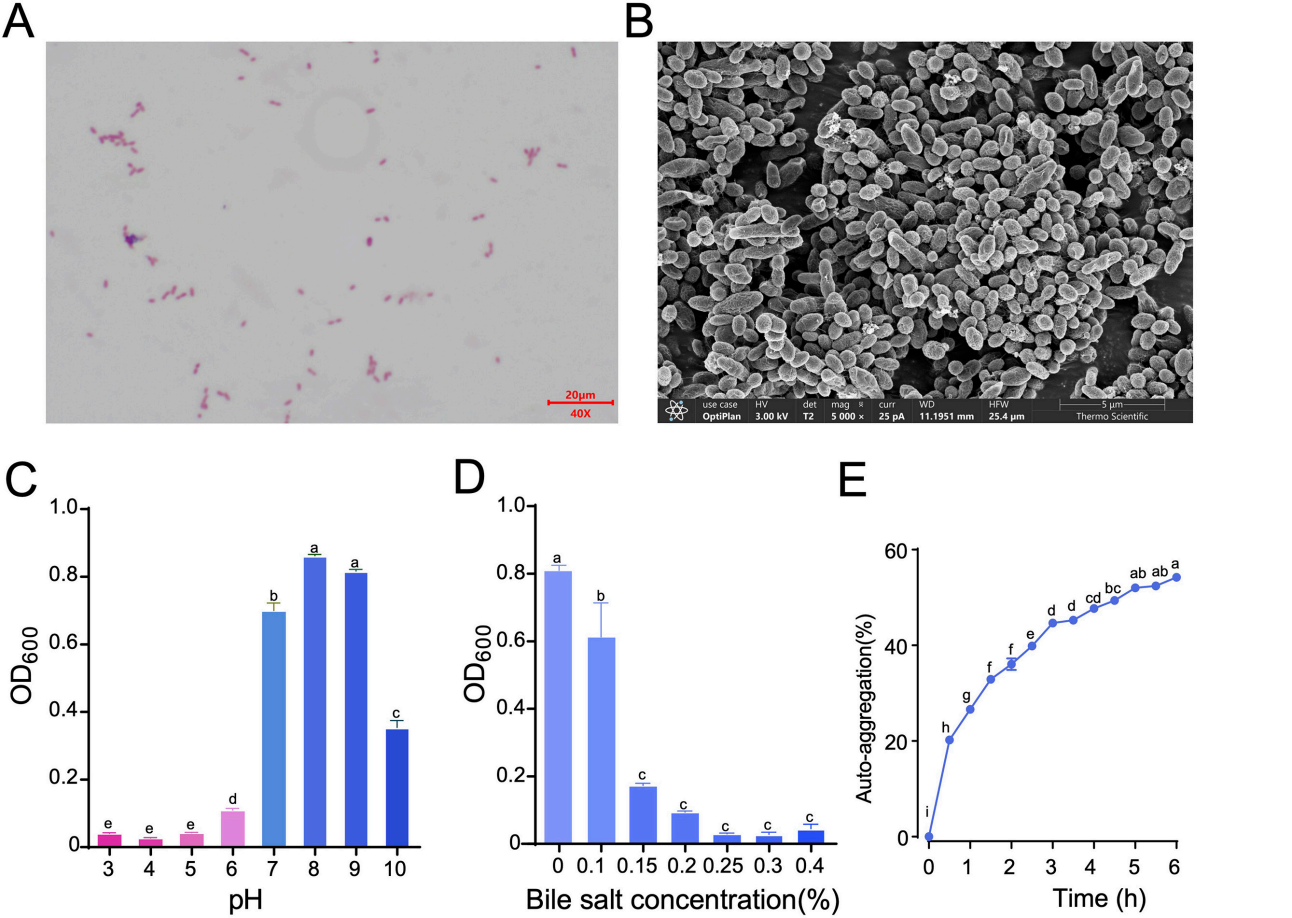
General probiotic properties of *Akkermansia muciniphila AKM Lab-01*. **(A)** Gram-stained images of AKM Lab-01; **(B)** Scanning electron microscope images of AKM Lab-01; **(C)** Growth of AKM Lab-01 at different pH values; **(D)** Bile salt tolerance of AKM Lab-01; **(E)** Auto-aggregation of AKM Lab-01. Data are presented as Mean ± SD, *n* = 3. Statistical analysis was performed using one-way ANOVA followed by Dunnett's multiple comparisons test. Bars not sharing a letter differ significantly (*p* < 0.05).

*A. muciniphila* AKM Lab-01 is a Gram-negative, strictly anaerobic, non-motile, and non-spore-forming bacterium that resides in the human gut (Figure 5A). Individual cells typically measure between 0.5 and 1.0 μm in length and can be observed singly, in pairs, in short chains, or in aggregates (Figures 5A, B). This strain exhibits a broad pH tolerance, with growth observed at pH levels ranging from 6 to 10 (Figure 5C). For the bile salt tolerance assay, AKM Lab-01 exhibited effective growth in media containing bile salt concentrations up to 0.25% (*p* < 0.05), indicating its favorable bile salts tolerance (Figure 5D). To effectively exert beneficial effects, probiotics must achieve adequate mass through aggregation, a trait highly desirable due to its impact on adhesion to intestinal epithelial cells (Gómez et al., 2016). AKM Lab-01 exhibits an auto-aggregation capacity of 54% within 6 h (Figure 5E). Collectively, these findings suggest that AKM Lab-01 possesses potential probiotic characteristics and is capable of effectively colonizing the gut.

### 3.5 *Akkermansia muciniphila* AKM Lab-01 exerts anti-obesity effects in HFD-induced obese mice

**Figure 6:**
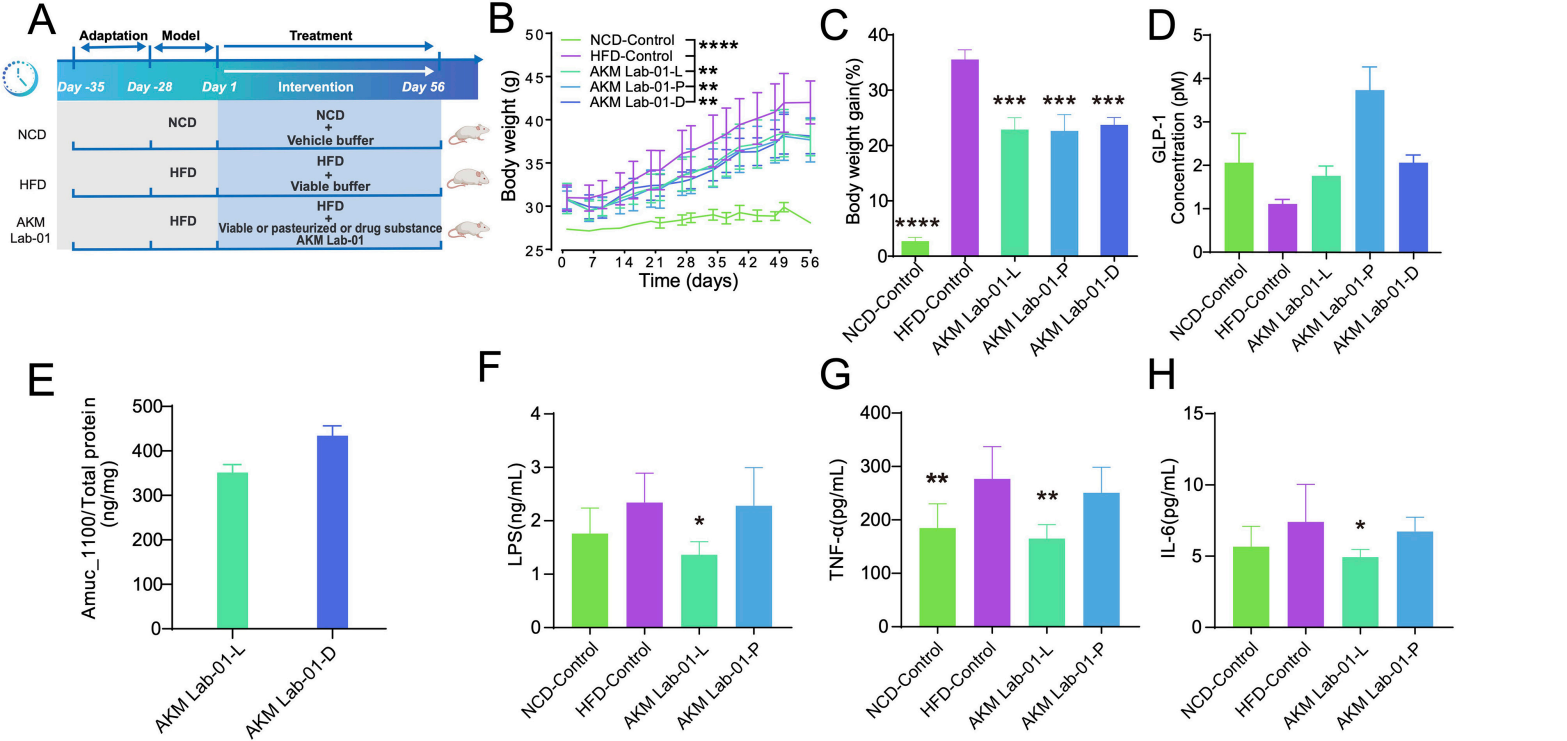
Viable, pasteurized and drug substance AKM Lab-01 exhibited anti-obesity effects in HFD mice. Five to six weeks old male C57B6/J were randomly assigned to five groups. The NCD group received a normal control diet (*n* = 8), while the remaining groups were fed HFD for 4 weeks to induce obesity. Subsequently, the obese mice underwent a 28-day treatment period, during which they were administered either PBS (HFD-Control, *n* = 8), viable AKM Lab-01 (AKM Lab-01-L, *n* = 8), pasteurized AKM Lab-01 (AKM Lab-01-P, *n* = 8), or pasteurized AKM Lab-01 drug substance (AKM Lab-01-D, *n* = 8). All treatments were delivered via oral gavage at a dose of 10^10^ TFU per mice. **(A)** Schematic diagram. **(B)** Curve graph of body weight. **(C)** Body weight gain (%) at the endpoint. **(D)** Serum levels of GLP-1 (active). **(E)** Quantitation of Amuc_1100 by mass spectrometry. Amuc_1100 protein was normalized to the total protein content extracted from the bacterial sample. **(F)** Serum LPS levels. **(G)** Serum TNF-α levels. **(H)** Serum IL-6 levels. Data are presented as Mean ± SD. Statistical analysis was performed by two-way *ANOVA* for the line chart **(B)** or one-way *ANOVA* for the bar chart combined with Dunnett's multiple comparisons test **(C, D, F–H)**. *ns*, not significant, *p < 0.05, **p < 0.01, ***p < 0.001, ****p < 0.0001.

To further evaluate the effects of different AKM Lab-01 formulations, we established a mild obesity model in mice by feeding them HFD for 4 weeks, followed by an 8-week intervention with three distinct formulations: viable (live) cells, pasteurized cells, and the drug substance form (Figure 6A). Our results revealed that all these three formulations-viable, pasteurized, and drug substance-effectively reduced weight gain in HFD-induced obese mice (Figures 6B, C). Previous studies have demonstrated that *A. muciniphila* combats obesity by regulating host energy metabolism through several mechanisms. Specifically, it thickens the intestinal mucus layer, thereby enhancing gut barrier function via the action of Amuc_1100 and reducing endotoxin entry into the bloodstream. This, in turn, lessens systemic inflammation (Mo et al., 2024). Additionally, *A. muciniphila* boosts the secretion of gut hormones like GLP-1 by producing SCFAs, particularly acetate and propionate. It also acts through the secreted protein P9 or the membrane protein Amuc_1100, thereby improving glucose tolerance and insulin sensitivity (Yoon et al., 2021). In the present study, we measured GLP-1 levels in blood samples from mice. The results showed that GLP-1 levels were significantly higher in the AKM Lab-01 treatment groups compared to the HFD-Control group (Figure 6D). Furthermore, we used mass spectrometry to quantify the content of Amuc_1100 protein in the different formulations of AKM Lab-01. The results demonstrated that the concentration of Amuc_1100 protein was 350 ng/mg in viable bacterial preparations and 450 ng/mg in the drug substance form (Figure 6E). Additionally, we assessed inflammation-related markers in mouse serum following AKM Lab-01 treatment. LPS levels were significantly reduced by viable AKM Lab-01 treatment compared to HFD controls, while pasteurized AKM Lab-01 showed a milder effect (Figure 6F). Similar reductions were observed for TNF-α and IL-6 (Figures 6G, H). These results indicate that AKM Lab-01 reduces endotoxin translocation and attenuates systemic inflammation.

To further assess the microbiome-mediated effects of AKM Lab-01, we conducted metagenomic sequencing comparing the HFD group and AKM Lab-01-treated group. While alpha diversity analysis revealed no significant changes in species diversity following AKM Lab-01 treatment (Supplementary Figure S4A), PCoA demonstrated significant alterations in gut microbiota composition (Supplementary Figure S4B). Further analysis of shifts in gut microbiota composition between the HFD group and the AKM Lab-01 treatment group is presented in Supplementary Dataset S5 and Supplementary Figures S4C, D. At the genus level, the abundance of *Anaerotruncus* and *Lawsonibacter*—genera associated with metabolic improvement (Lv et al., 2019; Liu et al., 2025)—was significantly increased after AKM Lab-01 treatment (*p* < 0.05). In contrast, the abundance of *Romboutsia, Allobaculum*, and *Eggerthella*—genera linked to obesity progression (Therdtatha et al., 2021; Zheng et al., 2021; Samudio-Cruz et al., 2025)—was markedly reduced following AKM Lab-01 supplementation. At the species level, *Lawsonibacter asaccharolyticus* (belonging to the genus *Lawsonibacter*) was notably increased, while *Romboutsia ilealis* (belonging to the genus *Romboutsia*) was significantly decreased in the AKM Lab-01 group (*p* < 0.05). We next conducted gene set enrichment analysis (GSEA) based on the KEGG pathway database to assess the functional impact of gut microbiota following AKM Lab-01 treatment. Notably, GSEA revealed a significant down-regulation in most disease-related pathways after treatment with AKM Lab-01, including Pathways of neurodegeneration – multiple diseases, arkinson disease, Alcoholic liver disease, and Alzheimer's disease (*p* < 0.05, FDR < 0.2, Supplementary Dataset S6 and Supplementary Figure S4E). These pathways have previously been linked to obesity (Neto et al., 2023; Zeng et al., 2025; Chiang and McCullough, 2014). Collectively, these results suggest that the amelioration of obesity-related phenotypes by AKM Lab-01 is associated with a remodeling of the gut microbiota in HFD-induced obese mice.

### 3.6 No adverse effects of AKM Lab-01 in 90-day toxicity study in mice

**Figure 7:**
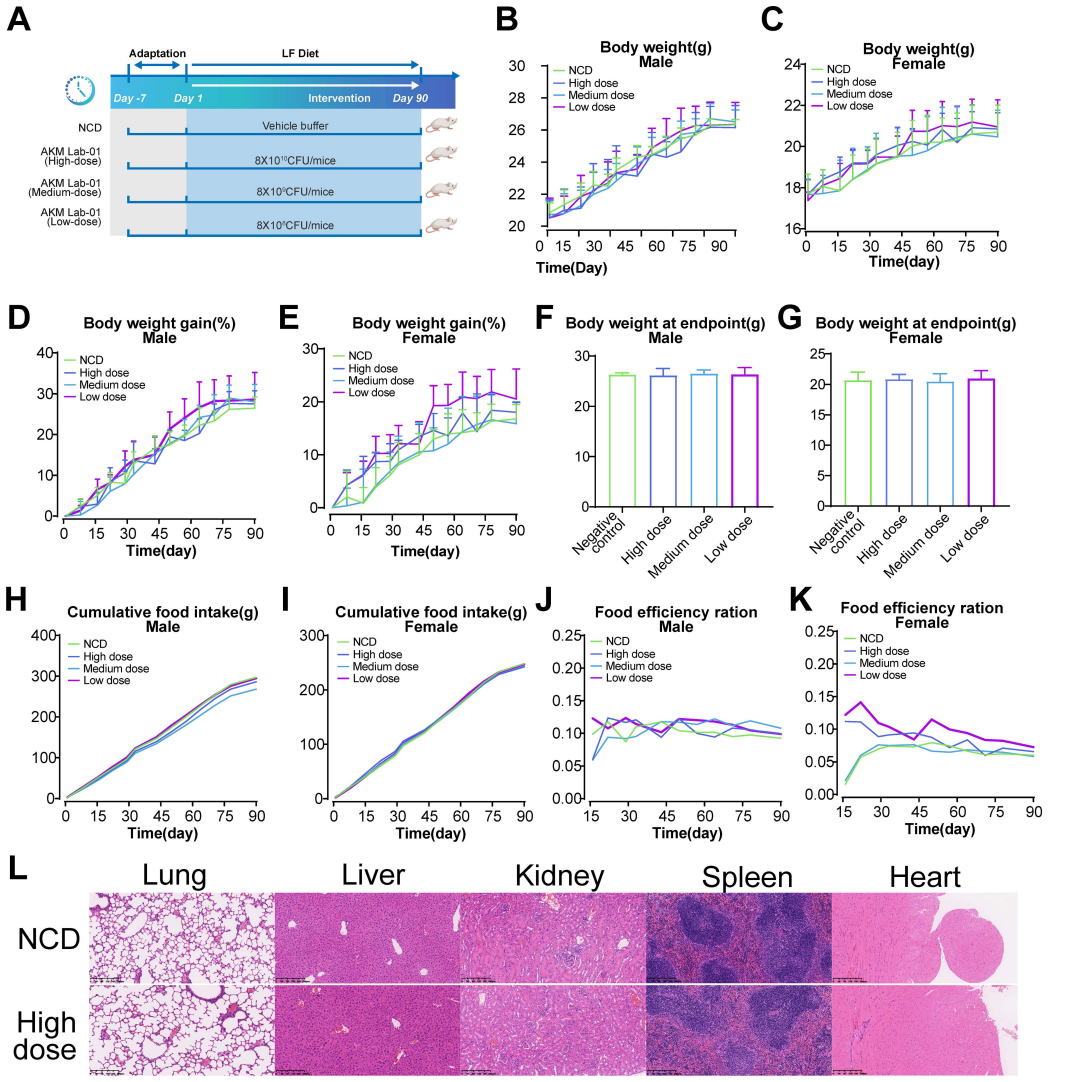
No adverse effects of AKM Lab-01 in 90-day toxicity study in mice. Six- to seven-week-old BALB/c mice (20 males and 20 females) were randomly divided into four groups (*n* = 10, half male and half female). The NCD group received only saline, whereas the other three groups administered AKM Lab-01 suspensions via oral gavage at daily dosages of 8.8 × 10^10^, 8.8 × 10^8^, and 8.8 × 108 TFU/mouse, respectively, for 90 consecutive days. **(A)** Schematic of experimental design. **(B, C)** Body weight. **(D, E)** Daily weight gain. **(F, G)** Final body weight. **(H, I)** Food consumption. **(J, K)** Food efficiency ratio. **(L)** Histopathological analysis. All data are expressed as Mean±SD and graphed and statistically analyzed using GraphPad Prism 10 software. Statistical analysis was performed by two-way *ANOVA* for the line chart **(B–E)** or one-way *ANOVA* for the bar chart combined with Dunnett's multiple comparisons test **(F, G)**. *ns*, not significant, not shown.

To evaluate the safety of *A. muciniphila* AKM Lab-01 drug substance (freeze-dried pasteurized AKM Lab-01), we conducted a 90-day dietary exposure study in mice. AKM Lab-01 drug substance was administered at daily dosages of 8.8 × 10^10^, 8.8 × 10^9^, and 8.8 × 108 TFU per mouse (Figure 7A). Throughout the study, no mortality or clinical signs were observed at any dosage level. There were no significant differences in body weight (Figures 7B, C), daily weight gain (Figures 7D, E), final body weight (Figures 7F, G), food consumption (Figures 7H, I), or food efficiency ratio between the treated and control groups (Figures 7J, K). Clinical chemistry analysis revealed that AST levels decreased in the low-dose group but increased in the high-dose group of female mice (Supplementary Table S1). Hematology and coagulation results were generally normal. However, a few non-dose-related changes were observed: a decrease in lymphocyte percentage at the high dose and in MCHC at the low dose, and an increase in neutrophil number at the low dose in male mice (Supplementary Table S2); an increase in basophil number, MCV, and MCH at the low dose in female mice (Supplementary Table S3). Organ weights were comparable between treated and control groups, except for a slight decrease in the weight of the liver, kidney, prostate glands, and epididymis at the medium dose. These changes were non-dose-related and attributed to individual variability rather than consumption of AKM Lab-01 (Supplementary Table S4). Histopathological analysis confirmed that AKM Lab-01 did not cause any abnormalities in the architecture of visceral organs, including the lung, liver, kidney, spleen, and heart (Figure 7L).

Therefore, under the study conditions and based on the toxicological parameters evaluated, no adverse effects were observed that could be attributed to the ingestion of AKM Lab-01 drug substance. Mice were able to safely consume AKM Lab-01 drug substance at concentrations up to 8 × 10^10^ TFU/day, corresponding to a daily dietary intake of 3.2 × 10^12^ TFU/kg body weight per day. Drawing on literature data (EFSA Panel on Nutrition et al., 2021) and the guidance of EFSA Scientific Committee (Committee, 2012), we applied an uncertainty factor of 200 [10 (interspecies variability) × 10 (intraspecies variability) × 2 (sub-chronic to chronic study duration)] to the NOAEL derived from the 90-day repeated-dose oral toxicity study in mice. Based on this analysis, we concluded that a daily intake of AKM Lab-01 drug substance at 1.12 × 10^12^ TFU is safe for an average 70 kg human, provided that the viable cell counts in AKM Lab- 01 drug substance remains below 10 TFU/g.

## 4 Discussion

Recent research has highlighted the pivotal role of gut microbiota in modulating energy metabolism, immune responses, and neurosignal transmission, suggesting its therapeutic potential for obesity and related metabolic disorders (Van Hul and Cani, 2023). Among these microbes, *A. muciniphila* has emerged as a promising candidate for regulating energy metabolism and improving obesity (Depommier et al., 2019; Zhang et al., 2025). Clinical cohort studies have revealed an inverse correlation between the abundance of *A. muciniphila* and the severity of obesity (Cani et al., 2022). Animal studies have demonstrated that *A. muciniphila* supplementation can reduce weight and fat accumulation and improve glucose tolerance in HFD-induced obese mice (Ioannou et al., 2025; Dao et al., 2016). A human intervention study further showed that pasteurized *A. muciniphila* improved insulin sensitivity, reduced insulin and cholesterol levels, and altered body fat distribution in overweight/obese individuals with insulin resistance (Depommier et al., 2019). Recently, another clinical trial investigated the effects of *A. muciniphila* supplementation (*A. muciniphila*-WST01) on patients with overweight or obese type 2 diabetes (T2D). The study found that *A. muciniphila*-WST01 effectively colonized the gut and improved metabolic parameters, including weight loss and reduced glycated hemoglobin (HbA1c), specifically in participants with low baseline levels of *A. muciniphila*. These findings suggest that the efficacy of *A. muciniphila* supplementation for metabolic benefits may depend on its initial abundance in the gut microbiota (Zhang et al., 2025).

For many years, *A. muciniphila* was the sole isolated representative of the *Verrucomicrobiota* phylum in the human gut. However, recent studies have identified only four to seven distinct phylogroups, likely due to the low abundance of these bacteria in samples and the limitations of current cultivation methods (Lv et al., 2022). Genomic analyses, however, suggest that there may be over 25 different *Akkermansia* species present in the human gut (Guo et al., 2017; Xing et al., 2019; Becken et al., 2021). In this study, we identified two putative novel species based on whole-genome sequencing and phylogenetic analysis of 26 representative strains isolated from healthy human fecal samples: one species showed close phylogenetic affiliation with *A. muciniphila*, while the other formed a distinct lineage in an outer branch. We also uncovered several instances of species misclassification. For example, *Akkermansia* sp. CAG:344 and *Akkermansia muciniphila* BSH01 were reclassified as *A. massiliensis*. Additionally, strains previously identified as *Candidatus Akkermansia timonensis* were reclassified as *Akkermansia biwaensis*. These findings suggest that the current taxonomy of the *Akkermansia* genus may need further optimization. Although newly discovered with few cultured strains, *A. massiliensis* exhibits more core gene families for key metabolic functions than *A. muciniphila* in our pan-genomic analysis. This suggests *A. massiliensis* has enhanced metabolic capabilities. However, the absence of key proteins like Amuc_1409 in *A. massiliensis* indicates that it might have a distinct mechanism.

*Akkermansia muciniphila* demonstrates strain-specific functional characteristics that elicit diverse host responses. For example, strains vary in their efficiency of utilizing human milk oligosaccharides (HMOs; Luna et al., 2022) and their capacity to synthesize vitamin B12 (Kirmiz et al., 2020). These differences can lead to distinct effects on gut health and metabolism, as observed in mouse models of inflammatory bowel diseases (Zhai et al., 2019; Liu et al., 2021) and obesity (Schneeberger et al., 2015; Jian et al., 2023). This underscores the critical need for comprehensive characterization of individual strains to elucidate their interactions and explore their potential applications. In this study, we identified *A. muciniphila* AKM Lab-01, a strain from our library collection, which significantly prevented weight gain in mice fed HFD and exhibited weight-reducing effects in obese mice, while also improving metabolic parameters. In addition, this strain possesses promising probiotic properties, including pH tolerance, bile salt resistance, and high auto-aggregation, which facilitate effective gut colonization. Furthermore, AKM Lab-01 enhanced GLP-1 secretion, modulated the gut microbiota, reduced endotoxin translocation and attenuated systemic inflammation. Notably, these beneficial effects were observed not only with live bacteria but also with pasteurized and freeze-dried powdered forms of AKM Lab-01. This suggests its potential for diverse product applications.

Probiotics, after extensive research and practical testing, have been shown to be highly safe and offer significant health benefits. Traditional probiotic strains, such as *Lactobacillus* and *Bifidobacterium*, have been used in fermented foods and dietary supplements for over a century. These strains have been proven to improve gut health, relieve constipation and diarrhea, and boost immunity (Kechagia et al., 2013). However, next-generation probiotics (NGPs), such as *A. muciniphila* and *Faecalibacterium prausnitzii*, regulate the host's physiological functions through specific mechanisms and can prevent or treat certain diseases. For example, *A. muciniphila* can improve metabolic syndrome and diabetes (Cani et al., 2022), while *F. prausnitzii* helps treat inflammatory bowel diseases (Martín et al., 2017). These probiotics have relatively clear mechanisms of action. However, they have a short history of use, mostly developed as microbiome-based drugs, with a few like *A. muciniphila* beginning to cross over into the food sector. Therefore, safety is also one of the important issues of public concern. In accordance with the guidelines from the US FDA and EFSA for toxicity testing of novel nonabsorbable food ingredients, Druart et al. conducted a comprehensive toxicological safety evaluation of pasteurized *A. muciniphila* (strain MucT, ATCC BAA-835). The evaluation included assessments of potential genotoxicity using bacterial reverse mutation and *in vitro* mammalian cell micronucleus tests, followed by a 90-day oral toxicity study to evaluate subchronic toxicity. The NOAEL was determined to be 1500 mg/kg body weight/day (equivalent to 9.6 × 10^10^ cells/kg body weight/day), which was the highest dose tested (Druart et al., 2021). Another study performed a comprehensive safety assessment for a novel and live strain of *A. muciniphila* (PROBIO), including its tolerance to gastrointestinal conditions, potential genotoxicity, antibiotic resistance, and acute and sub-chronic toxicity. The NOAEL for the 90-day study was concluded to be 9.2 × 10^9^ cells/kg body weight/day (the highest dose tested), corresponding to doses of 6.4 × 10^11^ viable bacteria for an average 70 kg human being (Ma et al., 2024). In our study, the safety assessment of freeze-dried pasteurized AKM Lab-01 through a 90-day dietary exposure study in mice revealed no adverse effects at high doses (up to 8 × 10^10^ TFU/day). This corresponds to a daily intake of 1 × 10^12^ TFU/day for an average human with a body weight of 70 kg. This finding underscores AKM Lab-01's potential as a promising probiotic for the prevention and treatment of obesity and related metabolic diseases.

In conclusion, our study provides compelling evidence of AKM Lab-01's efficacy and safety in modulating obesity and related metabolic disorders. Future research should focus on long-term clinical trials and further elucidation of its mechanisms of action to translate these findings into practical therapeutic strategies.

## 5 Limitations

It is important to acknowledge several limitations of our study. First, our findings are based on preclinical mouse models, and no human trial data are available—the efficacy and safety profile of AKM Lab-01 in mitigating obesity therefore requires rigorous validation in human cohorts. Notably, a clinical study investigating AKM Lab-01 for anti-hypercholesterolemia (NCT06974266) is currently ongoing, and future analyses of its outcomes may also provide insights into the strain's effects on metabolic conditions relevant to obesity. Second, host-microbe interactions exhibit inherent variability across human populations, driven by factors such as genetics, diet, and baseline microbiota; interspecies differences in gut microbial composition and host-microbe crosstalk may further impact disease progression and therapeutic responses, highlighting the need for future human-focused investigations to clarify AKM Lab-01′s mechanism of action in humans. In this study, we observed comparable weight-reducing effects between pasteurized and live bacterial preparations in obesity models, the differential effects of these two formulations in more complex host settings remain uncharacterized. Although pasteurized *A. muciniphila* (particularly the Amuc_1100 protein) is known to exert anti-obesity effects, live bacteria also secrete regulatory proteins (e.g., P9) that contribute to weight modulation—this intricate, multifactorial regulatory network requires further elucidation to clarify formulation-specific mechanisms. Additionally, there were some limitations in the experimental methods. For instance, the prevention and treatment models may differ in physiological states and mechanisms of intervention. Similarly, treatment models with different durations of model induction and intervention may also lead to variations in these aspects. Further repeated experiments are needed to verify these differences. Finally, functional data on microbiota composition after AKM Lab-01 intervention remain limited: while we observed alterations in microbiota taxa linked to obesity, comprehensive assessment of how these compositional shifts translate to functional changes (e.g., metabolic pathway activity, metabolite production) is lacking. Addressing these interconnected limitations will be critical to establishing a robust theoretical foundation for AKM Lab-01's future clinical applications.

## 6 Conclusion

The gut microbiota, particularly *A. muciniphila*, has emerged as a key player in regulating host metabolism and improving obesity. In this study, we isolated 222 strains of *Akkermansia* and conducted comprehensive genomic and functional analyses on selected strains. Eight *Akkermansia* species, including two potential novel species, were identified. Functional proteins, such as Amuc_1409, were conserved in *A. muciniphila* and closely related strains. Among these strains, AKM Lab-01 was identified and demonstrated significant efficacy in reducing weight gain and resisting HFD-induced obesity. After a 4-week intervention in obese mice, body weight was decreased by 10%, blood glucose and lipid levels were lowered, insulin resistance was improved, and indicators of metabolic syndrome were ameliorated. Enhanced expression of GLP-1 was observed and may contribute to the anti-obesity effects of AKM Lab-01. A 90-day dietary exposure study in mice showed no adverse effects from AKM Lab-01 drug substance ingestion, with a safe daily intake of up to 1 × 10^12^ TFU for humans. These findings highlight the potential of AKM Lab-01 as a promising probiotic for preventing and treating obesity.

## Data Availability

The genomic database supporting the comparative genomic and functional analyses in this article is described in Supplementary Dataset S1. The genome of AKM Lab-01 have been deposited in the NCBI database under BioProject accession number PRJNA1304677 (https://www.ncbi.nlm.nih.gov/bioproject/PRJNA1304677). The genome assembly accession number of AKM Lab-01 is JBQKMJ000000000, and the public repository URL address is https://www.ncbi.nlm.nih.gov/nuccore/JBQKMJ000000000.
