# Supplementary material for: Probiotic attributes and safety profile of AKM Lab-01: a novel Akkermansia muciniphila strain combating obesity and metabolic disorders in diet-induced obese mice
Source: Front Microbiol. 2026 Jan 6;16:1627870. doi: 10.3389/fmicb.2025.1627870 (PMC12815876; doi:10.3389/fmicb.2025.1627870)
Supplement: Supplementary file 7 [file Data_Sheet_1.docx]

**Supplementary Materials for**

Probiotic Attributes and Safety Profile of AKM Lab-01: A Novel *Akkermansia muciniphila* Strain Combating Obesity and Metabolic Disorders in Diet-Induced Obese Mice

Baojia Huang^1#^, Yibo Xian^1#^, Wenbin Xue^1^, Zilun Pu^1^, Ping Kong^1^, Peifen Li^1^, Yingying Zhao^1^, Lihong Tai^1^, Zhipeng Chen^1^, Zhou Lan^1^, Hong-Wei Liu^2^, Xianzhi Jiang^1^**^*^**, Amanda Juan Chen^1^**^*^**

^1^ Moon (Guangzhou) Biotech Co. Ltd., Huangpu District, Guangzhou, Guangdong, 510530, China

^2^ State Key Laboratory of Microbial Diversity and Innovative Utilization, Institute of Microbiology, Chinese Academy of Sciences, Beijing, P. R. China.

These authors contributed equally: Baojia Huang and Yibo Xian

**^*^Corresponding author**

Amanda Juan Chen, [email: chenj@moonbio.com](mailto:email:%20chenj@moonbio.com), Fax: 020-31603387, Telephone: +86-18910297089; Xianzhi Jiang, email: [jxz@moonbio.com](mailto:jxz@moonbio.com);

**This file includes**

Fig S1 Pan-genome accumulation curves

Fig S2 Cultivability assessment of potential *Akkermansia* strains

Fig S3 Genomic comparison between *A. muciniphila* AKM Lab-01 and reference *Akkermansia* strains

Fig S4 *A. muciniphila* AKM Lab-01 remodeled gut microbiota in HFD-induced obese mice

Fig S5 AKM Lab-01 and BAA-835 attenuated weight gain in HFD-induced obese mice

Table S1. Summary Data of Clinical Chemistry

Table S2. Summary Data of Hematology

Table S3. Summary Data of Coagulation

Table S4. Summary Data of Organ weight ratio at necropsy

Dataset 1 List of *Akkermansia* strains included in the bioinformatics analysis

Dataset 2 Genefamily COG annotation of *Akkermansia muciniphila* and *Akkermansia massiliensis*

Dataset 3 Bioinformatics predictions of virulence factors and COG analysis of potential *Akkermansia* strains

Dataset 4 List of single-copy core genes in *Akkermansia*

Dataset 5 Significant Differences in Microbial Abundance at the Species and Genus Levels

Dataset 6 GSEA of metagenomic sequencing

Fig S1 Pan-genome accumulation curves

A, Pan-genome accumulation curves of *Akkermansia muciniphila*;

B, Pan-genome accumulation curves of *Akkermansia massiliensis*.

Fig S2 Cultivability assessment of potential *Akkermansia* strains





Fig S3 Genomic comparison between *A. muciniphila* AKM Lab-01 and reference *Akkermansia* strains

A, Phylogenetic tree of *A. muciniphila* AKM Lab-01 and reference *Akkermansia* strains based on 16S rRNA gene sequences.

B, ANI (Average Nucleotide Identity) alignment between *A. muciniphila* AKM Lab-01 and reference *Akkermansia* strains.

C, COG comparison of *A. muciniphila* AKM Lab-01 and other *A. muciniphila* strains.


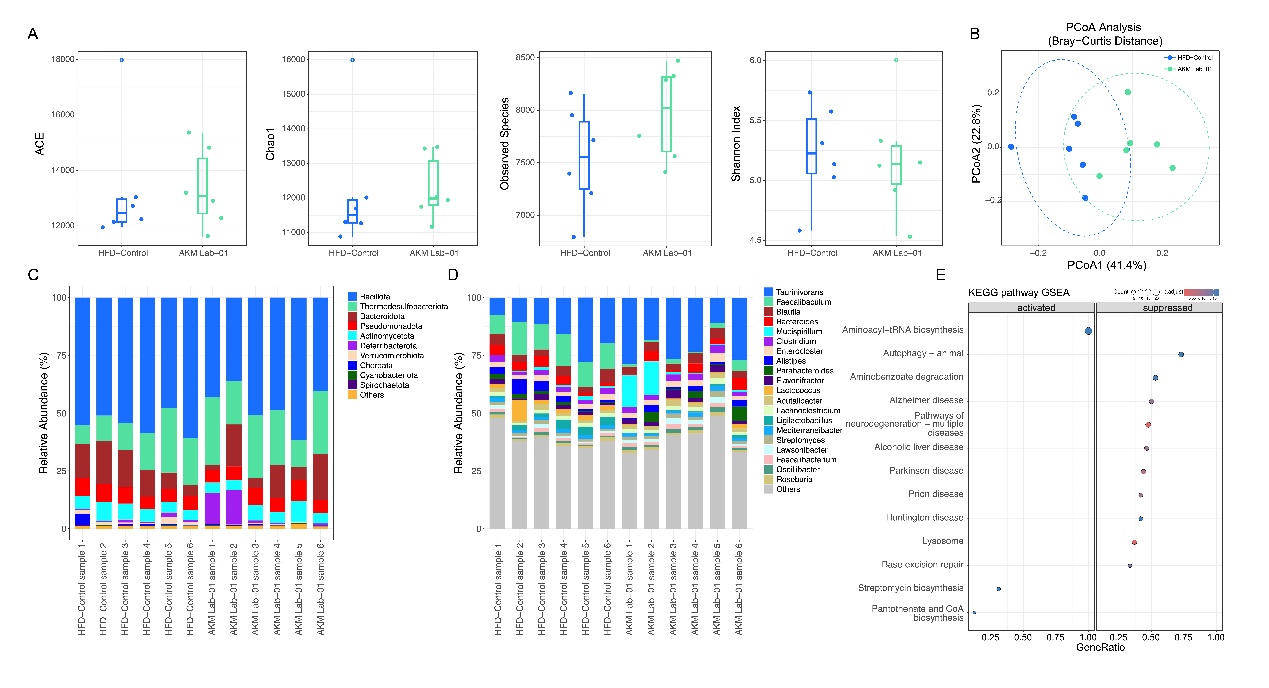
 Fig S4. *A. muciniphila* AKM Lab-01 remodeled gut microbiota in HFD-induced obese mice

A, α-diversity.

B, PCOA based on brav-curtis distance at the species level.

C-D, Relative abundances of taxonomic profiling at family level (C) and at genus level (D).

E, GSEA of metagenomic sequencing (*P* <0.05, FDR<0.2)

Fig S5. AKM Lab-01 and BAA-835 attenuated weight gain in HFD-induced obese mice

(A) Experimental timeline. (B) Body-weight curves during 28-day gavage. (C) Cumulative body-weight gain (%) on day 28. are presented as Mean±SEM; n = 6 per group. **, P < 0.01; *, P < 0.05 versus HFD-Control (one-way ANOVA followed by Tukey’s post-hoc test).

**Table S1 Summary Data of Clinical Chemistry**

| **Male** | **Control** | **AKM Lab-01-High** | | **AKMLab-01-Medium** | | **AKM Lab-01-Low** | |
| --- | --- | --- | --- | --- | --- | --- | --- |
|  | Mean±SD | Mean±SD | %Diff | Mean±SD | %Diff | Mean±SD | %Diff |
| ALT  (U/L) | 13.054±6.486 | 17.414±2.609 | -4.360 | 15.350±3.410 | -2.296 | 25.248±3.772 | -0.050 |
| AST  (U/L) | 62.534±8.901 | 59.114±11.402 | 3.420 | 59.092±17.107 | 3.442 | 69.196±10.717 | 4.248 |
| ALP  (U/L) | 59.012±4.622 | 66.062±10.473 | -7.050 | 59.802±4.283 | -0.790 | 63.936±10.094 | -3.504 |
| TP  (g/L) | 45.580±1.085 | 47.340±2.027 | -1.760 | 45.580±0.856 | 0.000 | 41.060±2.102 | 0.340 |
| ALB  (g/L) | 30.640±0.385 | 31.600±1.210 | -0.96 | 30.700±0.863 | -0.060 | 29.540±1.544 | 0.020 |
| T-Bil  (umol/L) | 0.720±0.268 | 0.860±0.261 | -0.140 | 0.800±0.245 | -0.080 | 0.320±0.110 | -0.080 |
| CHO  (mmol/L) | 2.182±0.105 | 2.176±0.092 | 0.006 | 2.262±0.076 | -0.080 | 1.522±0.104 | -0.120 |
| TG  (mmol/L) | 1.286±0.119 | 0.968±0.159 | 0.318 | 1.618±0.182 | -0.332 | 0.908±0.179 | -0.130 |
| HDL  (mmol/L) | 1.916±0.135 | 1.892±0.087 | 0.024 | 1.92±0.071 | -0.004 | 1.242±0.077 | -0.030 |
| LDL  (mmol/L) | 0.184±0.036 | 0.136±0.022 | 0.048 | 0.194±0.026 | -0.010 | 0.132±0.020 | 0.018 |
| UREA  (mmol/L) | 6.174±0.442 | 6.584±0.545 | -0.410 | 6.652±0.838 | -0.478 | 6.160±0.856 | 0.072 |
| CRE  (umol/L) | 8.580±1.787 | 10.000±0.738 | -1.420 | 9.780±1.361 | -1.200 | 9.980±1.401 | 0.260 |

| **Female** | **Control** | **AKM Lab-01-High** | | **AKM Lab-01-Medium** | | **AKM Lab-01-Low** | |
| --- | --- | --- | --- | --- | --- | --- | --- |
|  | Mean±SD | Mean±SD | %Diff | Mean±SD | %Diff | Mean±SD | %Diff |
| ALT  (U/L) | 22.178±1.863 | 26.355±2.687 ^a^ | -4.177 | 25.248±3.772 | -3.070 | 20.134±1.094 | 2.044 |
| AST  (U/L) | 66.936±12.540 | 79.3725±3.612 ^a^ | -12.440 **** | 69.196±10.717 | -2.260 | 55.786±13.651 | 11.150 **** |
| ALP  (U/L) | 72.646±7.728 | 67.858±8.563 | 4.788 | 63.936±10.094 | 4.979 | 69.206±6.586 | 3.440 |
| TP  (g/L) | 40.260±2.276 | 41.620±1.209 | -1.360 | 41.060±2.102 | -0.800 | 39.26±1.496 | 1.000 |
| ALB  (g/L) | 29.260±1.222 | 30.000±0.943 | -0.740 | 29.540±1.544 | -0.280 | 28.440±1.260 | 0.820 |
| T-Bil  (umol/L) | 0.62±0.277 | 0.54±0.305 | 0.080 | 0.32±0.110 | 0.300 | 0.204±0.152 | 0.380 |
| CHO  (mmol/L) | 1.54±0.138 | 1.54±0.148 | 0.000 | 1.522±0.104 | 0.018 | 1.484±0.048 | 0.056 |
| TG  (mmol/L) | 1.060±0.181 | 0.942±0.159 | 0.118 | 0.908±0.179 | 0.152 | 1.002±0.201 | 0.058 |
| HDL  (mmol/L) | 1.262±0.111 | 1.246±0.115 | 0.016 | 1.242±0.077 | 0.02 | 1.230±0.037 | 0.032 |
| LDL  (mmol/L) | 0.144±0.013 | 0.116±0.026 | 0.028 | 0.132±0.020 | 0.012 | 0.120±0.007 | 0.024 |
| UREA  (mmol/L) | 5.016±1.0160 | 5.764±1.381 | -0.748 | 6.160±0.856 | -1.144 | 6.174±0.539 | -1.158 |
| CRE  (umol/L) | 11.400±1.100 | 10.320±0.804 | 1.080 | 9.980±1.401 | 1.420 | 9.02±1.359 | 2.380 |

All data are expressed as Mean±SD. Statistical analysis using GraphPad software (version 10.2.3) was performed by multiple comparisons using two-way ANOVA followed by Tukey's multiple comparison test. Ns: not significant, not showed; *: p < 0.05. All the indicators except ALT/AST in AKM Lab-01-High group in the female group were measured from five mice.

a, Data of 4 mice were included in analysis.

**Table S2 Summary Data of Hematology**

| **Male** | **Control** | **AKM Lab-01-High** | | **AKM Lab-01-Medium** | | **AKM Lab-01-Low** | |
| --- | --- | --- | --- | --- | --- | --- | --- |
|  | Mean±SD | Mean±SD | %Diff | Mean±SD | %Diff | Mean±SD | %Diff |
| WBC  (109/L) | 4.348±  0.729 | 3.888±1.987 | -10.580 | 4.262±0.993 | -1.978 | 5.010±1.163 | 15.225 |
| Neu  (%) | 19.720±  4.501 | 23.740±1.252 | 20.385 | 21.320±5.959 | 8.114 | 22.460±2.912 | 13.895 |
| Lym  (%) | 73.500±  3.533 | 68.500±3.605 | -6.803 * | 70.900±5.381 | -3.537 | 70.760±2.990 | -3.728 |
| Mon  (%) | 4.960±  1.743 | 5.720±3.187 | 15.323 | 4.320±1.307 | -12.903 | 5.080±0.920 | 2.419 |
| Eos  (%) | 0.900±  0.552 | 1.600±1.384 | 77.778 | 1.360±0.744 | 51.111 | 1.080±0.510 | 20.000 |
| Bas  (%) | 0.920±  0.295 | 0.440±0.321 | -52.174 | 2.100±2.534 | 128.261 | 0.620±0.303 | -32.609 |
| Neu#  (109/L) | 0.836±  0.110 | 0.930±0.475 | 11.244 | 0.878±0.209 | 5.024 | 1.106±0.123 | 32.297 * |
| Lym#  (109/L) | 3.216±  0.671 | 2.710±1.503 | -15.734 | 3.060±0.891 | -4.851 | 3.572±0.971 | 11.070 |
| Mon#  (109/L) | 0.222±  0.105 | 0.182±0.058 | -18.018 | 0.174±0.029 | -21.622 | 0.254±0.087 | 14.414 |
| Eos#  (109/L) | 0.038±  0.028 | 0.046±0.033 | 21.053 | 0.056±0.036 | 47.368 | 0.050±0.028 | 31.579 |
| Bas#  (109/L) | 0.036±  0.013 | 0.020±0.019 | -44.444 | 0.094±0.119 | 161.111 | 0.028±0.013 | -22.222 |
| RBC  (1012/L) | 5.334±  0.285 | 6.100±0.518 | 14.361 | 5.814±0.552 | 8.999 | 6.040±0.542 | 13.236 |
| HGB  (g/L) | 120.400±5.771 | 135.400±9.940 | 12.458 | 128.400±13.164 | 6.645 | 134.800±12.834 | 11.960 |
| HCT  (%) | 21.380±  0.942 | 24.260±2.063 | 13.471 | 22.960±2.257 | 7.390 | 24.120±2.398 | 12.816 |
| MCV  (fL) | 40.040±  0.513 | 39.820±0.249 | -0.549 | 39.500±0.505 | -1.349 | 39.880±0.390 | -0.400 |
| MCH  (pg) | 22.580±  0.311 | 22.220±0.421 | -1.594 | 22.080±0.286 | -2.214 | 22.300±0.200 | -1.240 |
| MCHC  (g/L) | 563.400±2.608 | 558.600±11.261 | -0.852 | 559.000±5.916 | -0.781 | 559.000±3.162 | -0.781 * |
| RDW-CV  (%) | 15.68±  0.942 | 16.08±0.798 | 2.551 | 15.620±0.455 | -0.383 | 15.660±0.456 | -0.128 |
| RDW-SD  (fL) | 26.360±  1.991 | 26.700±1.362 | 1.290 | 25.820±0.576 | -2.049 | 26.080±0.890 | -1.062 |
| PLT  (109/L) | 516.400±53.388 | 565.200±49.505 | 9.450 | 584.600±68.690 | 13.207 | 537.800±35.245 | 4.144 |
| MPV  (fL) | 5.580±  0.228 | 5.480±0.084 | -1.792 | 5.500±0.122 | -1.434 | 5.600±0.141 | 0.358 |
| PDW | 15.320±  0.148 | 15.220±0.084 | -0.653 | 15.160±0.114 | -1.044 | 15.380±0.084 | 0.392 |
| PCT  (%) | 0.288±  0.038 | 0.310±0.024 | 7.564 | 0.321±0.034 | 11.312 | 0.302±0.015 | 4.650 |

| **Female** | **Control** | **AKM Lab-01-High** | | **AKM Lab-01-Medium** | | **AKM Lab-01-Low** | |
| --- | --- | --- | --- | --- | --- | --- | --- |
|  | Mean±SD | Mean±SD | %Diff | Mean±SD | %Diff | Mean±SD | %Diff |
| WBC  (109/L) | 8.024±3.514 | 3.582±0.441 | -55.359 | 4.626±1.031 | -42.348 | 7.026±2.129 | -12.438 |
| Neu  (%) | 22.060±7.751 | 26.680±4.801 | 20.943 | 18.540±4.589 | -15.957 | 16.440±1.286 | -25.476 |
| Lym  (%) | 71.120±8.975 | 55.600±14.211 | -21.822 | 75.940±3.745 | 6.777 | 74.720±2.533 | 5.062 |
| Mon  (%) | 3.560±1.566 | 6.960±2.911 | 95.506 | 3.160±1.489 | -11.236 | 4.560±1.698 | 28.090 |
| Eos  (%) | 2.420±1.377 | 3.580±1.961 | 47.934 | 1.080±0.349 | -55.372 | 1.500±0.596 | -38.017 |
| Bas  (%) | 0.840±0.230 | 7.180±9.609 | 754.7619 | 1.280±0.920 | 52.381 | 2.780±0.760 | 230.952 |
| Neu#  (109/L) | 1.904±1.469 | 0.970±0.243 | -49.0546 | 0.856±0.296 | -55.042 | 1.146±0.319 | -39.811 |
| Lym#  (109/L) | 5.550±2.082 | 1.988±0.531 | -64.1802 | 3.514±0.796 | -36.6847 | 5.262±1.660 | -5.189 |
| Mon#  (109/L) | 0.280±0.207 | 0.242±0.091 | -13.5714 | 0.150±0.078 | -46.429 | 0.314±0.098 | 12.143 |
| Eos#  (109/L) | 0.226±0.170 | 0.126±0.074 | -44.2478 | 0.048±0.022 | -78.7611 | 0.104±0.050 | -53.982 |
| Bas#  (109/L) | 0.064±0.036 | 0.256±0.350 | 300.000 | 0.058±0.052 | -9.375 | 0.200±0.093 | 212.500 * |
| RBC  (1012/L) | 5.394±2.431 | 6.038±0.759 | 11.939 | 6.122±0.215 | 13.496 | 5.848±0.426 | 8.417 |
| HGB  (g/L) | 115.000±51.347 | 132.000±14.612 | 14.783 | 135.800±6.340 | 18.087 | 131.800±8.468 | 14.609 |
| HCT  (%) | 21.260±9.719 | 23.520±2.382 | 10.630 | 24.520±1.167 | 15.334 | 23.820±1.669 | 12.041 |
| MCV  (fL) | 39.320±0.377 | 39.000±1.000 | -0.814 | 40.060±0.577 | 1.882 | 40.740±0.207 | 3.611 * |
| MCH  (pg) | 21.300±0.453 | 21.920±0.311 | 2.911 | 22.180±0.342 | 4.131 | 22.540±0.416 | 5.822 * |
| MCHC  (g/L) | 542.600±12.857 | 561.000±8.803 | 3.391 | 553.600±2.881 | 2.027 | 553.800±8.585 | 2.064 |
| RDW-CV  (%) | 16.800±3.169 | 18.480±1.537 | 10.000 | 16.060±0.416 | -4.405 | 16.080±0.665 | -4.286 |
| RDW-SD  (fL) | 27.760±5.195 | 30.100±1.580 | 8.429 | 26.920±0.942 | -3.026 | 27.420±1.252 | -1.225 |
| PLT  (109/L) | 516.200±159.035 | 527.400±86.028 | 2.170 | 455.200±37.413 | -11.8171 | 416.000±68.615 | -19.411 |
| MPV  (fL) | 5.220±0.687 | 5.520±0.277 | 5.747 | 5.640±0.152 | 8.045977 | 5.660±0.182 | 8.429 |
| PDW | 14.880±0.626 | 15.420±0.164 | 3.629 | 15.400±0.071 | 3.495 | 15.420±0.228 | 3.629 |
| PCT  (%) | 0.279±0.131 | 0.292±0.061 | 4.953 | 0.256±0.019 | -8.040 | 0.2354±0.038 | -15.506 |

All data are expressed as Mean±SD. Statistical analysis using GraphPad software (version 10.2.3) was performed by multiple comparisons using two-way ANOVA followed by Tukey's multiple comparison test. ns: not significant, not shown; *: p < 0.05.

**Table S3 Summary Data of Coagulation**

| **Male** | **Control** | **AKM Lab-01-High** | | **AKM Lab-01-Medium** | | **AKM Lab-01-Low** | |
| --- | --- | --- | --- | --- | --- | --- | --- |
|  | Mean±SD | Mean±SD | %Diff | Mean±SD | %Diff | Mean±SD | %Diff |
| PT (s) | 14.860±0.527 | 14.740±2.678 | 0.120 | 15.700±2.094 | -0.840 | 13.440±1.828 | 1.420 |
| APTT (s) | 62.260±5.587 | 59.920±6.365 | 2.340 | 63.860±6.510 | -1.6 | 61.620±4.260 | 0.640 |
| TT (s) | 157.000±102.733 | 88.580±77.469 | 68.420 | 159.640±79.110 | -2.640 | 126.420±38.768 | 30.580 |
| FIB (g/L) | 2.724±2.358 | 0.840±0.202 | 1.884 | 0.616±0.209 | 2.108* | 0.790±0.395 | 1.934 |

| **Female** | **Control** | **AKM Lab-01-High** | | **AKM Lab-01-Medium** | | **AKM Lab-01-Low** | |
| --- | --- | --- | --- | --- | --- | --- | --- |
|  | Mean±SD | Mean±SD | %Diff | Mean±SD | %Diff | Mean±SD | %Diff |
| PT (s) | 58.100±47.134 | 35.800±32.330 | 22.300 | 17.200±3.166 | 40.900 | 15.560±2.220 | 42.540 |
| APTT (s) | 51.180±28.666 | 62.800±10.127 | -11.620 | 61.300±12.189 | -10.120 | 66.980±11.438 | -15.800 |
| TT (s) | 77.880±77.007 | 165.640±98.168 | -87.760 | 245.160±290.120 | -167.300 | 91.680±56.905 | -13.800 |
| FIB (g/L) | 4.966±9.172 | 0.94±0.413 | 4.026 | 1.824±0.576 | 3.142 | 1.534±0.687 | 3.432 |

All data are expressed as Mean±SD and statistically analyzed using GraphPad software (version 10.2.3). Statistical analysis is performed by multiple comparisons using one-way ANOVA followed by Dunnett multiple comparison test. ns: not significant, not shown; *: p < 0.05.

**Table S4 Summary data of organ weight ratio at necropsy**

| **Male** | **Control** | **AKM Lab-01-High** | | **AKM Lab-01-Medium** | | **AKM Lab-01-Low** | |
| --- | --- | --- | --- | --- | --- | --- | --- |
|  | Mean±SD | Mean±SD | %Diff | Mean±SD | %Diff | Mean±SD | %Diff |
| Brain | 1.594±0.169 | 1.583±0.132 | -0.007 | 1.706±0.077 | 0.070 | 1.627±0.070 | 0.021 |
| Thymus  gland | 0.140±0.037 | 0.144±0.022 | 0.033 | 0.148±0.039 | 0.061 | 0.156±0.039 | 0.117 |
| Liver | 3.975±0.059 | 3.463±0.287 | -0.129** | 3.493±0.149 | -0.121* | 3.430±0.316 | -0.137** |
| Spleen | 0.282±0.031 | 0.293±0.023 | 0.038 | 0.276±0.047 | -0.021 | 0.284±0.025 | 0.006 |
| Kidney | 1.553±0.146 | 1.367±0.113 | -0.120 | 1.335±0.236 | -0.140 | 1.373±0.068 | -0.116 |
| Adrenal  gland | 0.020±0.0039 | 0.022±0.003 | 0.108 | 0.053±0.087 | 1.650 | 0.012±0.004 | -0.393 |
| Prostate glands | 0.177±0.022 | 0.176±0.035 | -0.008 | 0.118±0.004 | -0.331 | 0.157±0.055 | -0.113 |
| Testis | 0.808±0.070 | 0.766±0.042 | -0.052 | 0.610±0.269 | -0.245 | 0.716±0.041 | -0.113 |
| Epididymis | 0.284±0.014 | 0.268±0.029 | -0.055 | 0.181±0.095 | -0.362 | 0.228±0.020 | -0.196 |
| Heart | 0.563±0.028 | 0.592±0.039 | 0.05095 | 0.513±0.064 | -0.089 | 0.534±0.051 | -0.0519 |

| **Female** | **Control** | **AKM Lab-01-High** | | **AKM Lab-01-Medium** | | **AKM Lab-01-Low** | |
| --- | --- | --- | --- | --- | --- | --- | --- |
|  | Mean±SD | Mean±SD | %Diff | Mean±SD | %Diff | Mean±SD | %Diff |
| Brain | 1.958±0.049 | 2.070±0.035 | 0.057 | 2.019±0.049 | 0.031 | 2.083±0.123 | 0.064* |
| Thymus gland | 0.166±0.020 | 0.178±0.006 | 0.073 | 0.151±0.009 | -0.087 | 0.148±0.008 | -0.109 |
| Liver | 3.469±0.133 | 3.589±0.155 | 0.035 | 3.305±0.134 | -0.047 | 3.298±0.104 | -0.049 |
| Spleen | 0.384±0.041 | 0.363±0.026 | -0.054 | 0.352±0.036 | -0.083 | 0.382±0.067 | -0.005 |
| Kidney | 1.263±0.060 | 1.137±0.090 | -0.100* | 1.093±0.066 | -0.134** | 1.149±0.042 | -0.090* |
| Adrenal gland | 0.039±0.009 | 0.040±0.004 | 0.029 | 0.040±0.012 | 0.014 | 0.045±0.011 | 0.154 |
| Ovary | 0.082±0.006 | 0.050±0.020 | -0.386 | 0.072±0.036 | -0.126 | 0.075±0.006 | -0.087 |
| Heart | 0.517±0.051 | 0.518±0.081 | 0.0035 | 0.513±0.061 | -0.006 | 0.523±0.091 | 0.012 |

All data are expressed as Mean±SD and statistically analyzed using GraphPad software (version 10.2.3). Statistical analysis is performed by multiple comparisons using one-way ANOVA followed by Dunnett multiple comparison test. ns: not significant, not showed; *: p < 0.05, **: p < 0.01, ***: p < 0.001, ****: p < 0.0001.
